# Design, synthesis, HER2 inhibition and anticancer evaluation of new substituted 1,5-dihydro-4,1-benzoxazepines

**DOI:** 10.1080/14756366.2021.1948841

**Published:** 2021-07-12

**Authors:** Olga Cruz-López, Matilde Ner, Francho Nerín-Fonz, Yaiza Jiménez-Martínez, David Araripe, Juan A. Marchal, Houria Boulaiz, Hugo Gutiérrez-de-Terán, Joaquín M. Campos, Ana Conejo-García

**Affiliations:** a Department of Medicinal and Organic Chemistry, Faculty of Pharmacy, University of Granada, Granada, Spain; b Biosanitary Institute of Granada (ibs.GRANADA), SAS-University of Granada, Granada, Spain; c Department of Cell and Molecular Biology, Uppsala University, Uppsala, Sweeden; d Biopathology and Medicine Regenerative Institute, University of Granada, Granada, Spain; e Excellence Research Unit “Modeling Nature” (MNat), Department of Human Anatomy and Embryology, University of Granada, Granada, Spain

**Keywords:** Antitumour, pyroptosis, HER2 receptor, molecular modelling, benzoxazepines

## Abstract

A series of 11 new substituted 1,5-dihydro-4,1-benzoxazepine derivatives was synthesised to study the influence of the methyl group in the 1-(benzenesulphonyl) moiety, the replacement of the purine by the benzotriazole bioisosteric analogue, and the introduction of a bulky substituent at position 6 of the purine, on the biological effects. Their inhibition against isolated HER2 was studied and the structure–activity relationships have been confirmed by molecular modelling studies. The most potent compound against isolated HER2 is **9a** with an IC_50_ of 7.31 µM. We have investigated the effects of the target compounds on cell proliferation. The most active compound (**7c**) against all the tumour cell lines studied (IC_50_ 0.42–0.86 µM) does not produce any modification in the expression of pro-caspase 3, but increases the caspase 1 expression, and promotes pyroptosis.

## Graphical Abstract

**Figure:**
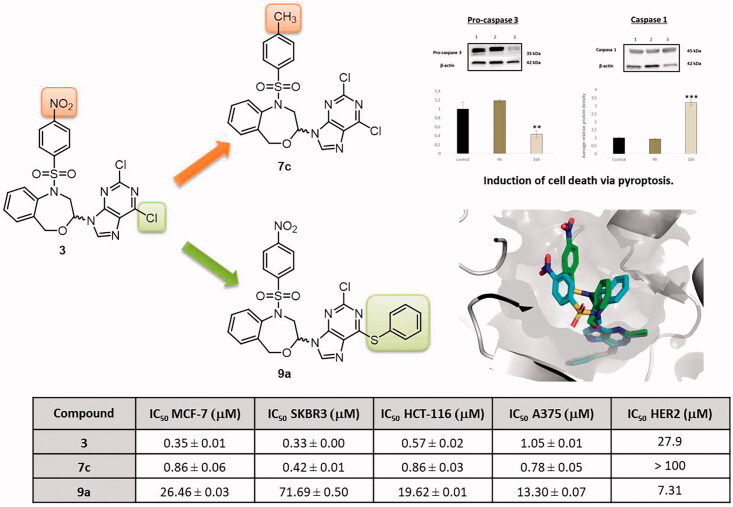

## Introduction

Cancer continues to be one of the leading causes of morbidity and mortality in the world, with approximately 18.1 million new cases in 2018. Population estimates indicate that the number of new cases will increase in the next two decades to 29.5 million a year in 2040. Breast cancer is the most commonly occurring cancer in women and the second most common cancer overall (11.6% of the total cases in 2018) followed by colorectal cancer (10.2%)^1^. Although its incidence is much lower, melanoma also deserves special attention since it represents one of the fastest growing types of cancer, with advanced metastatic forms presenting high mortality rates^2^^,^^3^.

The inherent heterogeneity of this disease, the dose-limiting adverse effects due to the unspecificity of classic chemotherapeutic agents and the appearance of chemoresistance are some of the drawbacks in current cancer treatments. To tackle these limitations, the development of new anticancer drugs is among the top priorities in medicinal chemistry.

Some of the most important signalling pathways involved in cancer are those linked to cell proliferation, differentiation and survival, all of which are promising targets for new antitumour drugs. As a result, the human epidermal growth factor receptor 2 (HER2) is often overexpressed in a variety of epithelial cancers in humans^4^^,^^5^. About 20% of breast cancer patients over-express HER2^6^. HER2 is an essential protein for cell division and normal cell growth, its abnormal overexpression is related to cancerous processes associated with poor prognosis and an overall survival decrease in patients^7^.

**Figure 1.**
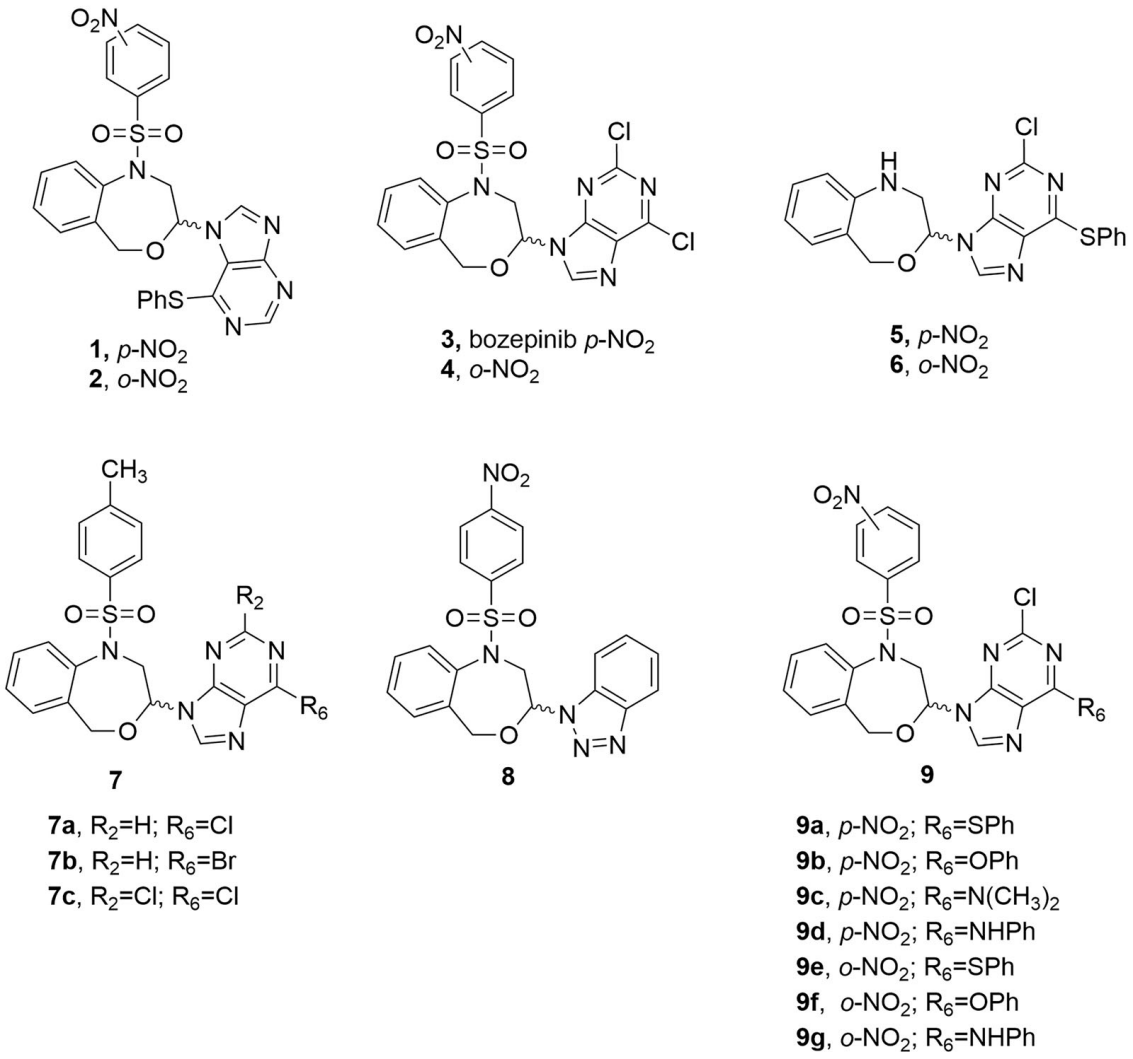
Chemical structures of the previously reported 4,1-benzoxazepine-purines (**1**-**6**) and molecular modifications employed for the design of the target molecules (**7**–**9**).

We previously described a series of 4,1-benzoxazepine-purines as potent antitumour compounds^8–10^. Compounds **1** and **2**, with a phenylthio substituent in position 6 of the purine moiety (Figure 1) exhibited *in vitro* antiproliferative activities in the micromolar range (0.86 µM and 2.59 µM, respectively) against the MCF-7 human breast cancer cell line^9^. Later on, the *N*-9 regioisomers **3** and **4** (Figure 1) derived from the 2,6-dichloropurine also displayed relevant activities (0.355 µM and 0.383 µM, respectively), unlike analogues **5** and **6** which do not present the nitrobenzenesulphonyl group (Figure 1) notably decreased the cytotoxic effect of the compounds, with IC_50_ values of 9.71 µM (**5**) and 13.85 µM (**6**) against the MCF-7 cell line. (*RS*)-2,6-dichloro-9-[1-(*p*-nitrobenzenesulfonyl)-1,2,3,5-tetrahydro-4,1-benzoxazepine-3-yl]-9*H*-purine (**3**, also named bozepinib, Figure 1) was the most potent and selective antitumour compound developed in our group^11^. The role of protein kinase-R (PKR) as a biological target for bozepinib in the apoptosis of breast and colon cancer cells was demonstrated. Furthermore, the inhibition of HER2, c-Jun N-terminal kinase (JNK) and extracellular signal-regulated protein kinases (ERKs), as well as antiangiogenic and antimigration activity, as well as the antitumour and antimetastatic activity *in vivo*, were reported^11^^,^^12^. Very recently, we have demonstrated that bozepinib treatment was able to reduce glioblastoma cell viability by apoptosis and autophagy induction, without any change in interference on cell cycle progression or Akt activation^13^. We also showed that one cycle of treatment with bozepinib selects resistant cells increasing the percentage of CD133^+^ and the activity of NF-ƙB cells and, however, two cycles of treatment eliminate the resistant cells^13^.

Taken together, these compounds may serve as prototypes for the development of even more potent structures endowed with a new mechanism of action.

Thus, we present here a new series of substituted 1,5-dihydro-4,1-benzoxazepines with the aim to develop antitumour agents. First, the methyl group was chosen to replace the nitro one of bozepinib in the present study. The different steps of the biotransformations that produce a primary amine from an aromatic nitro compound involve the nitro radical ion, the nitroso derivative, the nitroxyl radical, the hydroxylamine and then the primary amine. Each of these different intermediates could contribute to the toxicity^14^. Then, the substitution of the nitro (**3**, Figure 1) by a methyl group (**7**, Figure 1) eliminates the above-mentioned toxic metabolites. In addition, this methyl group may be oxidised to an alcohol and further to a carboxylic acid, favouring its elimination from the body.

Purines are the most abundant and stable *N*-heterocyclic compounds found in nature. Many drug development programmes have focussed on structural modifications of natural purines, especially using bioisosteric ring systems. Thus, we also envisioned the benzotriazole analogue **8** for their close relationship with the purine fragment of compound **3**. The benzotriazole can be considered as a privileged structure for its several pharmacological activities^15^ being useful as scaffold for the design of new antitumour compounds^16^.

Finally, since the substituent size at the position 6 of the purine and the presence of the nitrobenzenesulphonyl group appear to be the essential characteristics for the antiproliferative effect, we decided to design molecules that combined both structural features (**9**, Figure 1) to complete the target compounds of the present study.

## Experimental

### Chemistry

#### General

Melting points were taken in open capillaries on a Stuart Scientific SMP3 electrothermal melting point apparatus and are uncorrected. Analytical thin layer chromatography (TLC) was performed using Merck Kieselgel 60 F_254_ aluminium plates and visualised by UV light or iodine. All evaporations were carried out *in vacuo* in a Büchi rotary evaporator and the pressure controlled by a Vacuubrand CVCII apparatus. Merck silicagel 60 (Merck, Kenilworth, NJ) with a particle size of 0.040–0.063 mm (230–400 mesh ASTM) was used for flash chromatography. Small-scale microwave-assisted synthesis was carried out in an Initiator 2.0 single-mode microwave instrument producing controlled irradiation at 2.450 GHz (Biotage AB, Upsala, Sweden). Reaction time refers to hold time at 110 °C or 160 °C, not to total irradiation time. These parameters were established following the basic principles of microwave assisted organic synthesis. The temperature was measured with an IR sensor on the outside of the reaction vessel. Nuclear magnetic resonance (NMR) spectra were recorded on a 500 MHz ^1^H and 125 MHz ^13 ^C NMR Varian Direct Drive spectrometer at ambient temperature. Chemical shifts (*δ*) are quoted in parts per million (ppm) and are referenced to the residual solvent peak. Spin multiplicities are given as s (singlet), bs (broad singlet), d (doublet), dd (double doublet), ddd (double double doublet), pt (pseudo triplet) and m (multiplet). *J* values are given in Hz. Electrospray ionisation (ESI-TOF) mass spectra were carried out on a Waters LCT Premier Mass Spectrometer, respectively. Anhydrous CH_2_Cl_2_ and CH_3_CN were purchased from VWR International Eurolab (Barcelona, Spain). Anhydrous conditions were performed under argon. All reagents were purchased from Aldrich. Compounds **3**^10^, **4**^10^, **10**^17^ and **12**^17^ were synthesised as previously reported.

### 3-Methoxy-1-(*p*-methylbenzenesulfonyl)-1,2,3,5-tetrahydro-4,1-benzoxazepine (11)

#### Method a

To a solution of **10**^17^ (250 mg, 1.38 mmol) and *p*-methylbenzenesulphonyl chloride (532 mg, 2.79 mmol) in anhydrous CH_2_Cl_2_ (4.2 ml), was added triethylamine (0.582 ml, 4.18 mmol) at 0 °C under argon. The reaction mixture was microwave-irradiated at 110 °C for 30 min, then poured into water and extracted with CH_2_Cl_2_. The organic phase was washed with brine, dried over Na_2_SO_4_ and evaporated. The residue was purified by flash chromatography using EtOAc/hexane 1/7 as eluent.

#### Method b

To a solution of **10**^17^ (500 mg, 2.79 mmol) and pyridine (0.449 ml, 5.58 mmol) in anhydrous CH_2_Cl_2_ (25 ml), *p*-methylbenzenesulphonyl chloride (797 mg, 4.18 mmol) was added at 0 °C under argon. The reaction mixture was stirred for 3 days at room temperature, then poured into water and extracted with CH_2_Cl_2_. The organic phase were washed with 1 N HCl aq., water and brine, dried over Na_2_SO_4_ and evaporated. The residue was purified by flash chromatography using EtOAc/hexane 1/7 as eluent.

White solid, method a: 185 mg (40%), and method b: 620 mg (67%), mp: 106–107 °C. ^1^H NMR (CDCl_3_): *δ* (ppm) 7.56 (d, *J* = 8.3 Hz, 2H), 7.50 (d, *J* = 7.7 Hz, 1H), 7.30–7.14 (m, 5H), 4.66 (pt, *J* = 3.9, *J* = 3.8 Hz, 1H), 4.43 (d, *J* = 13.6 Hz, 1H), 4.10 (d, *J* = 13.6 Hz, 1H), 3.94–3.63 (bs, 2H), 3.38 (s, 3H), 2.40 (s, 3H). ^13 ^C NMR (CDCl_3_): 144.35 (C), 140.53 (C), 138.64 (C), 138.20 (C), 130.15 (CH) (× 2), 130.04 (CH), 129.72 (CH), 129.66 (CH), 128.69 (CH), 128.51 (CH) (× 2), 101.68 (CH), 65.11 (CH_2_), 56.18 (CH_3_), 54.37 (CH_2_), 22.54 (CH_3_). HRMS (ESI-TOF) (*m/z*) calcd. for C_17_H_19_NO_4_SNa (M + Na)^+^ 356.0933, found 356.0932.

### General procedure for the synthesis of 7a, 7b, 7c and 8

A suspension of **11** or **12**^17^ (1 equiv) and the corresponding purine derivative or benzotriazole (2.5 equiv) in anhydrous acetonitrile (5 ml/mmol) was prepared under argon and cooled to 0 °C and TMSCl (4 equiv), HMDS (4 equiv) and a 1 M solution of SnCl_4_ (4 equiv) in CH_2_Cl_2_ were subsequently added. The temperature was allowed to rise to 10 °C before microwave irradiating at 160 °C for 5 min. The reaction was quenched by cooling (ice/water bath) and by the addition of water. The pH was fixed to 7–8 with a saturated NaHCO_3_ solution and the aqueous phase extracted with EtOAc. The combined organic layers were dried (Na_2_SO_4_), filtered and evaporated. **7a–7c** were purified by flash chromatography using EtOAc/hexane 1/5 as eluent. Compound **8** was obtained by washing the residue with CH_2_Cl_2_ and diethyl ether and the solid obtained was filtered.

### 6-Chloro-9-[1-(*p*-methylbenzenesulfonyl)-1,2,3,5-tetrahydro-4,1-benzoxazepine-3-yl]-9*H*-purine (7a)

White solid, 25 mg (12%), mp: 178–180 °C. ^1^H NMR (CDCl_3_, 400 MHz): *δ* (ppm) 8.81 (s, 1H, H2_pur_), 8.19 (s, 1H), 7.76 (d, *J* = 8.0 Hz, 2H), 7.38–7.34 (m, 6H), 6.14 (d, *J* = 9.7 Hz, 1H), 4.78–4.68 (m, 2H, H), 4.56 (d, *J* = 13.6 Hz, 1H), 3.68 (dd, *J* = 14.8, 10.0 Hz, 1H), 2.46 (s, 3H). ^13 ^C NMR (CDCl_3_, 75 MHz): 152.64 (CH), 151.67 (C), 151.11 (C), 144.70 (C), 143.06 (CH), 139.56 (C), 138.04 (C), 137.12 (C), 130.39 (CH) (×2), 130.14 (CH), 130.04 (CH), 129.22 (CH), 128.88 (CH), 127.62 (CH) (×2), 84.63 (CH), 71.81 (CH_2_), 54.52 (CH_2_), 21.88 (CH_3_). HRMS (ESI-TOF) (*m/z*) calcd. for C_21_H_19_N_5_O_3_SCl (M + H)^+^ 456.0897, found 456.0908.

### 6-Bromo-9-[1-(*p*-methylbenzenesulfonyl)-1,2,3,5-tetrahydro-4,1-benzoxazepine-3-yl]-9*H*-purine (7b)

White solid, 15 mg (10%), mp: 169–171 °C. ^1^H NMR (CDCl_3_, 400 MHz): *δ* (ppm) 8.80 (s, 1H), 8.23 (s, 1H), 7.80 (d, *J* = 8.2 Hz, 2H, Hab), 7.41–7.37 (m, 6H), 6.16 (dd, *J* = 9.9, 1.4 Hz, 1H, H3), 4.82–4.72 (m, 2H), 4.57 (d, *J* = 13.6 Hz, 1H), 3.70 (dd, *J* = 14.8, 10.0 Hz, 1H), 2.50 (s, 3H). ^13 ^C NMR (CDCl_3_, 75 MHz): 153.73 (CH), 151.01 (C), 145.86 (C), 144.08 (CH), 140.72 (C), 139.19 (C), 138.28 (C), 131.55 (CH) (×2), 131.30 (CH), 131.20 (CH), 130.38 (CH), 130.04 (CH), 128.78 (CH) (×2), 85.80 (CH), 72.98 (CH_2_), 55.68 (CH_2_), 23.04 (CH_3_). HRMS (ESI-TOF) (*m/z*) calcd. for C_21_H_19_N_5_O_3_SBr (M + H)^+^ 500.0392, found 500.0367.

### 2,6-Dichloro-9-[1-(*p*-methylbenzenesulfonyl)-1,2,3,5-tetrahydro-4,1-benzoxazepine-3-yl]-9*H*-purine (7c)

White solid, 49.5 mg (28%), mp: 193–195 °C. ^1^H NMR (CDCl_3_, 400 MHz): *δ* (ppm) 8.17 (s, 1H), 7.82 (d, *J* = 8.3 Hz, 2H), 7.45–7.32 (m, 6H), 6.01 (dd, *J* = 10.0, 1,8 Hz, 1H), 4.73–4.69 (m, 2H), 4.56 (d, *J* = 13.6 Hz, 1H), 3.60 (dd, *J* = 14.9, 10.0 Hz, 1H), 2.48 (s, 3H). ^13^C NMR (CDCl_3_, 75 MHz): 153.29 (C), 152.12 (C), 151.99 (C), 144.61 (C), 143.28 (CH), 139.27 (C), 137.57 (C), 136.47 (C), 130.69 (C), 130.30 (CH) (×2), 129.88 (CH), 129.84 (CH), 129.30 (CH), 128.61 (CH), 127.49 (CH) (×2), 84.03 (CH), 71.72 (CH_2_), 54.08 (CH_2_), 21.68 (CH_3_). HRMS (ESI-TOF) (*m/z*) calcd. for C_21_H_18_N_5_O_3_SCl_2_ (M + H)^+^ 490.0507, found 490.0491.

### 2-[1-(*p*-Nitrobenzenesulfonyl)-1,2,3,5-tetrahydro-4,1-benzoxazepine-3-yl]-2*H*-benzotriazole (8)

White pale solid, 38 mg (31%), mp: 209–211 °C. ^1^H NMR (DMSO-d_6_, 400 MHz,): *δ* (ppm) 8.43 (dd, *J* = 7.9, 2.0 Hz, 2H), 8.24 (dd, *J* = 7.9, 2.0 Hz, 2H), 8.13 (d, *J* = 8.4 Hz, 1H), 8.03 (d, *J* = 8.4 Hz, 1H), 7.64 (ddd, *J* = 8.1, 7.0, 0.8 Hz, 1H), 7.52–7.46 (m, 2H), 7.44–7.38 (m, 2H), 7.22–7.17 (m, 1H), 6.58 (dd, *J* = 9.9, 2.1 Hz, 1H), 4.98–4.83 (m, 3H), 4.26 (dd, *J* = 15.1, 9.9 Hz, 1H). ^13 ^C NMR (DMSO-d_6_, 100 MHz) *δ* 150.65 (C), 146.18 (C), 145.54 (C), 139.36 (C), 137.53 (C), 132.89 (C), 129.94 (CH), 129.20 (CH) (×2), 128.91 (CH), 128.68 (CH) (×2), 127.43 (CH), 125.53 (CH) (×2), 125.25 (CH), 119.84 (CH), 111.67 (CH), 86.40 (CH), 69.70 (CH_2_), 53.50 (CH_2_). HRMS (ESI-TOF) (*m/z*) calcd. for C_21_H_18_N_5_O_5_S (M + H)^+^ 452.1029, found 452.1027.

### General procedure for the synthesis of 9a and 9e (A)

To a solution of **1** or **2** (1.0 equiv) in DMF (5 ml/mmol), K_2_CO_3_ (1.6 equiv) and thiophenol (0.8 equiv) were added at room temperature and stirred for 1 h. The reaction mixture was concentrated and the residue was dissolved in CH_2_Cl_2_, washed with water and brine, dried (Na_2_SO_4_), filtered and evaporated. The crude products were purified by flash chromatography using EtOAc/hexane 3/1 as eluent.

### 2-Chloro-6-phenylthio-9-[1-(*p*-nitrobenzenesulfonyl)-1,2,3,5-tetrahydro-4,1-benzoxazepine-3-yl]-9*H*-purine (9a)

White solid, 37 mg (72%), mp: 183–185 °C. ^1^H NMR (CDCl_3_, 400 MHz): *δ* (ppm) 8.44 (d, *J* = 8.5 Hz, 2H), 8.15 (d, *J* = 8.5 Hz, 2H), 8.03 (s, 1H), 7.67–7.61 (m, 2H), 7.50–7.46 (m, 3H), 7.46–7.32 (m, 4H), 5.94 (d, *J* = 9.3 Hz, 1H), 4.74 (t, *J* = 13.5 Hz, 2H), 4.55 (d, *J* = 13.5 Hz, 1H), 3.64 (dd, *J* = 14.2, 7.1 Hz, 1H). ^13 ^C NMR (CDCl_3_, 100 MHz): 163.54 (C), 154.25 (C), 150.63 (C), 149.37(C), 146.18 (C), 141.02 (CH), 138.69 (C), 136.49 (C), 135.44 (CH) (×2), 130.27 (CH), 130.08 (CH), 129.91 (CH), 129.38 (CH) (×2), 129.20 (CH), 129.07 (CH), 129.03 (CH) (×2), 126.29 (C), 124.99 (CH) (×2), 84.31 (CH), 71.82 (CH_2_), 54.55 (CH_2_). HRMS (ESI-TOF) (*m*/*z*) calcd. for C_26_H_20_ClN_6_O_5_S_2_ (M + H)^+^ 595.0625, found 595.0652.

### 2-Chloro-6-phenylthio-9-[1-(*o*-nitrobenzenesulfonyl)-1,2,3,5-tetrahydro-4,1-benzoxazepine-3-yl]-9*H*-purine (9e)

White solid, 20 mg (35%), mp: 212–214 °C. ^1^H NMR (CDCl_3_, 400 MHz): *δ* (ppm) 8.14 (dd, *J* = 7.1, 1.7 Hz, 1H), 8.03 (s, 1H), 7.85–7.74 (m, 3H), 7.68–7.62 (m, 2H), 7.51–7.43 (m, 3H), 7.42–7.38 (m, 2H), 7.36–7.30 (m, 1H), 7.16 (d, *J* = 7.7 Hz, 1H), 6.00 (dd, *J* = 10.0, 1.8 Hz, 1H), 5.01 (d, *J* = 13.7 Hz, 1H), 4.82 (d, *J* = 13.7 Hz, 1H), 4.72 (dd, J = 15.0, 1.8 Hz, 1H), 3.81 (dd, *J* = 10.0, 15.0 Hz, 1H). ^13^C NMR (CDCl_3_, 100 MHz): 163.23 (C), 154.06 (C), 149.48 (C), 147.76 (C), 141.03 (CH), 138.76 (C), 137.35 (C), 135.30 (CH) (×2), 134.53 (CH), 133.54 (C), 132.35 (CH), 132.31 (CH), 130.17 (CH), 129.69 (CH), 129.62 (CH), 129.22 (CH) (× 2), 129.10 (CH), 128.71 (CH), 126.42 (C), 124.63 (CH), 84.51 (CH), 71.81 (CH_2_), 54.65 (CH_2_). HRMS (ESI-TOF) (*m*/*z*) calcd. for C_26_H_20_ClN_6_O_5_S_2_ (M + H)^+^ 595.0625, found 595.0652.

### General procedure for the synthesis of 9b, 9c, 9d, 9f and 9g (B)

A solution of **3** or **4** (1.0 equiv), phenol or aniline (2.0 equiv) and Et_3_N (2.0 equiv) in DMF (5 ml/mmol) was stirred at 80 °C (3 h for **9d** and **9g**, 6h for **9f**, 20h for **9b** and **9c**). The reaction mixture was concentrated and the residue was dissolved in CH_2_Cl_2_, washed with water and brine, dried (Na_2_SO_4_), filtered and evaporated. The residue was purified by flash chromatography using EtOAc/hexane 1/1 for **9b** and **9c**, 2/1 for **9d** and **9g** and 4/1 for **9f** as eluent.

### 2-Chloro-6-phenoxy-9-[1-(*p*-nitrobenzenesulfonyl)-1,2,3,5-tetrahydro-4,1-benzoxazepine-3-yl]-9*H*-purine (9b)

Orange solid, 24 mg (48%), mp: 210–212 °C. ^1^H NMR (CDCl_3_, 400 MHz): *δ* (ppm) 8.45 (d, *J* = 8.7 Hz, 2H), 8.16 (d, *J* = 8.7 Hz, 2H), 8.07 (s, 1H), 7.48–7.35 (m, 6H), 7.33–7.25 (m, 3H), 5.98 (d, *J* = 9.4 Hz, 1H), 4.77 (d, *J* = 13.8 Hz, 2H); 4.56 (d, *J* = 13.8 Hz, 1H), 3.68 (dd, *J* = 14.7, 10.0 Hz, 1H). ^13 ^C-NMR (CDCl_3_, 100 MHz): 160.41 (C), 153.66 (C), 152.11 (C), 150.65 (C), 146.25 (C), 140.83 (C), 138.71 (C), 136.52 (C), 130.28 (CH), 130.12 (CH), 129.71 (CH) (×2), 129.24 (CH), 129.10 (CH), 129.01 (CH) (×2), 126.17 (CH), 125.00 (CH) (×2), 121.53 (CH) (×2), 84.38 (CH), 71.83 (CH_2_), 54.53 (CH_2_). HRMS (ESI-TOF) (*m*/*z*) calcd. for C_26_H_20_ClN_6_O_6_S (M + H)^+^ 579.0854, found 579.0817.

### 2-Chloro-6-dimethylamino-9-[1-(*p*-nitrobenzenesulfonyl)-1,2,3,5-tetrahydro-4,1-benzoxazepine-3-yl]-9*H*-purine (9c)

Yellow solid, 12 mg (26%), mp: 203–205 °C. ^1^H NMR (CDCl_3_, 400 MHz): *δ* (ppm) 8.46 (d, *J* = 8.2 Hz), 8.19 (d, *J* = 8.2 Hz), 7.91 (s, 1H), 7.49–7.41 (m, 2H), 7.39 (t, *J* = 7.2 Hz, 1H); 7.34 (d, *J* = 7.2 Hz, 1H), 5.88 (d, *J* = 6.7 Hz, 1H), 4.78–4.69 (m, 2H), 4.44 (d, *J* = 13.7 Hz, 1H), 4.12 (bs, 3H, N-CH_3_), 3.78 (bs, 3H, N-CH_3_), 3.55 (dd, *J* = 15.0, 10.1 Hz, 1H). ^13^C-NMR (CDCl_3_, 100 MHz): 154.72 (C), 153.59(C), 150.61 (C), 150.24(C), 146.17(C), 138.69(C), 136.32 (C), 135.95 (CH), 130.20 (CH), 130.00 (CH), 129.48 (CH), 129.19 (CH) (×2), 129.08 (CH), 125.02 (CH) (×2), 116.55 (C), 83.90 (CH), 71.87 (CH_2_), 54.52 (CH_2_), 44.11 (CH_3_), 43.53 (CH_3_). HRMS (ESI-TOF) (*m*/*z*) calcd. for C_22_H_21_ClN_7_O_5_S (M + H)^+^ 530.1013, found 530.1012.

### 2-Chloro-6-anilino-9-[1-(*p*-nitrobenzenesulfonyl)-1,2,3,5-tetrahydro-4,1-benzoxazepine-3-yl]-9*H*-purine (9d)

Yellow solid, 44 mg (85%), mp: 182–183 °C. ^1^H NMR (CDCl_3_, 400 MHz): *δ* (ppm) 9.87 (bs, 1H), 8.54 (bs, 1H); 8.46 (d, *J* = 8.0 Hz, 2H), 8.20 (d, *J* = 8.0 Hz, 2H), 7.84 (d, *J* = 8.0 Hz, 2H), 7.46–734 (m, 6H), 7.19 (t, *J* = 7.3 Hz, 1H), 6.05 (s, 1H), 4.90 (d, *J* = 11.6 Hz, 1H), 4.83 (d, *J* = 13.3 Hz, 1H), 4.67 (d, *J* = 13.3 Hz, 1H), 3.58 (bs, 1H). ^13^C-NMR (CDCl_3_, 100 MHz): 157.03 (C), 150.71 (C), 150.34 (C), 148.24 (C), 145.86 (C), 138.78 (C), 137.41 (C), 136.22 (C), 135.95 (C), 130.33 (CH), 130.30 (CH), 129.28 (CH), 129.23 (CH) (×2), 129.16 (CH) (×2), 129.02 (CH), 125.15 (CH), 125.06 (CH) (×2), 120.72 (CH) (×2), 85.12 (CH), 72.14 (CH_2_), 54.22 (CH_2_). HRMS (ESI-TOF) (*m*/*z*) calcd. for C_26_H_21_ClN_7_O_5_S (M + H)^+^ 578.1013, found 578.0991.

### 2-Chloro-6-phenoxy-9-[1-(*o*-nitrobenzenesulfonyl)-1,2,3,5-tetrahydro-4,1-benzoxazepine-3-yl]-9*H*-purine (9f)

Yellowish solid, 38 mg (47%), mp: 142–144 °C. ^1^H NMR (CDCl_3_, 400 MHz): *δ* (ppm) 8.11 (dd, *J* = 1.6, 7.3 Hz, 1H), 8.05 (s, 1H) 7.82–7.72 (m, 3H), 7.46–7.34 (m, 4H), 7.34–7.20 (m, 4H), 7.12 (d, *J* = 7.7 Hz, 1H), 6.03 (dd, *J* = 10.0, 1.8 Hz, 1H), 5.00 (d, *J* = 13.6 Hz, 1H), 4.80 (d, *J* = 13.6 Hz, 1H), 4.72 (dd, *J* = 14.9, 1.8 Hz, 1H), 3.81 (dd, *J* = 14.9, 10.0 Hz, 1H). ^13^C-NMR (CDCl_3_, 100 MHz): 160.41 (C), 153.59 (C), 153.38 (C), 152.21 (C), 147.92 (C), 141.20 (CH), 138.91 (C), 137.61 (C), 134.74 (CH), 133.73 (C), 132.55 (CH), 132.49 (CH), 130.38 (CH), 129.82 (CH), 129.74 (CH) (×2), 129.33 (CH), 128.85 (CH), 126.14 (CH), 124.84 (CH), 121.61 (CH) (×2), 120.72 (C), 84.83 (CH), 71.99 (CH_2_), 54.86 (CH_2_). HRMS (ESI-TOF) (*m*/*z*) calcd. for C_26_H_20_ClN_6_O_6_S (M + H)^+^ 579.0854, found 579.0817.

### 2-Chloro-6-anilino-9-[1-(*o*-nitrobenzenesulfonyl)-1,2,3,5-tetrahydro-4,1-benzoxazepine-3-yl]-9*H*-purine (9g)

Yellowish solid, 43 mg (82%), mp: 152–157 °C. ^1^H NMR (CDCl_3_, 400 MHz): *δ* (ppm) 10.05 (bs, 1H), 8.54 (bs, 1H), 8.23–8.19 (m, 1H), 7.87 (d, *J* = 7.9 Hz, 2H), 7.85–7.80 (m, 2H), 7.79–7.75 (m, 1H), 7.43-7.31 (m, 5H), 7.21–7.15 (m, 2H), 6.08 (d, *J* = 9.3 Hz), 5.06 (d, *J* = 13.5 Hz, 1H), 4.89 (dd, *J* = 14.6, 5.1 Hz, 2H), 3.72 (dd, *J* = 14.6, 9.3 Hz, 1H). ^13^C-NMR (CDCl_3_, 100 MHz): 157.11 (C), 150,31 (C), 148.26 (C), 147.83 (C), 138.86 (C), 137.50 (C), 136.69 (C), 136.03 (C), 134.79 (CH), 133.38 (C), 132.61 (CH), 132.57 (CH), 130.29 (CH), 129.97 (CH), 129.28 (CH), 129.17 (CH) (×2), 128.89 (CH), 125.06 (CH), 124.83 (CH), 121.98 (CH), 120.81 (CH) (×2), 85.32 (CH), 72.29 (CH_2_), 54.69 (CH_2_). HRMS (ESI-TOF) (*m*/*z*) calcd. for C_26_H_21_ClN_7_O_5_S (M + H)^+^ 578.1013, found 578.0991.

### Biology

#### HER2 inhibition assay

The IC_50_ profile of the target compounds was determined using isolated HER2. IC_50_ values were measured by testing 10 concentrations (1 × 10 ^−4 ^M to 3 × 10 ^−9 ^M) of each compound in singlicate. A radiometric protein kinase assay (33PanQinase® Activity Assay) was used for measuring the kinase activity of HER2. The assay was performed in 96-well FlashPlatesTM from PerkinElmer (Boston, MA) in a 50 µl reaction volume. The assay contained 70 mM HEPES-NaOH pH 7.5, 3 mM MgCl_2_, 3 mM MnCl_2_, 3 µM Na-orthovanadate, 1.2 mM DTT, 50 µg/ml PEG20000, ATP (corresponding to the apparent ATP-Km of the kinase), [γ-^33^P]-ATP (approx. 7.2 × 1005 cpm per well), HER2, and the target compound solutions in DMSO (the final DMSO concentration in the reaction cocktails was 1% in all cases). The reaction cocktails were incubated at 30 °C for 60 min. The reaction was halted with 50 µl of 2% (v/v) H_3_PO_4_, plates were aspirated and washed two times with 200 µl 0.9% (w/v) NaCl. Incorporation of ^33^P_i_ was determined with a microplate scintillation counter (Microbeta, Wallac, Waltham, MA).

#### Cell culture

MCF-7, HCT-116, A-375 and SKBR cells were grown at 37 °C in an atmosphere containing 5% CO_2_, with Dulbecco’s modified Eagle Medium (DMEM) (Gibco, Grand Island, NY) supplemented with 10% heat-inactivated foetal bovine serum (FBS) (Gibco), 2% l-glutamine, 2.7% sodium bicarbonate, 1% Hepes buffer, 40 mg/L gentamicin and 500 mg/L ampicillin.

#### Drug treatment

Compounds were dissolved in DMSO and stored at −20 °C. For each experiment, the stock solutions were further diluted in medium to obtain the desired concentrations. The final solvent concentration in cell culture was ≤ 0.1% v/v of DMSO, a concentration without any effect on cell replication. Parallel cultures of MCF-7, HCT-116, A375 and SKBR-3 cells in medium with DMSO were used as controls.

#### Proliferation assays

The effect of the compounds on cell viability was assessed using the sulphorhodamine-B colorimetric assay. Cells suspension (1 × 10^3^ cells/well) were seeded onto 24-well plates and incubated for 24 h. The cells were then treated with different concentrations of drugs in their respective culture medium and maintained with the treatment for 3 days. Three days later, wells were aspirated and fresh medium and drug were added. Then cell cultures were maintained for 3 additional days. Thereafter, a Titertek Multiscan (Flow, Irvine, CA) at 492 nm was used. The linearity of the SRB assay was evaluated with a cell number for each cell stock before each cell growth experiment. The IC_50_ values were calculated from semi-logarithmic dose–response curves by linear interpolation. All the experiments were plated in triplicate wells and were carried out twice.

#### Protein analysis

We performed a western blot to study differences in protein expression after treatment with 5 µM of **7c**.

SKBR cells seeded on 6-well plates were treated during 4 h and 16, lysed using RIPA Lysis Buffer System (Santa Cruz Biotechnology, Santa Cruz, CA) and quantified by Pierce™ BCA Protein Assay Kit (Thermo Fisher Scientific, Waltham, MA). Western blot was realised using 4× Laemmli sample buffer (Bio-Rad, Hercules, CA) and separated by SDS-polyacrylamide gel electrophoresis (SDS–PAGE) and then, transferred to PVDF membranes. Blocking was realised with milk in PBS 5%.

The following primary antibodies were used to detect protein expression,: caspase 3: ref-9662S (Cell Signalling, Beverly, MA), caspase 1(14F468): sc-56036 (Santa Cruz Biotechnology, Santa Cruz, CA), cytochrome-c (A-8): sc-13156 (Santa Cruz Biotechnology, Santa Cruz, CA), Bax(B-9): sc-7480 (Santa Cruz Biotechnology, Santa Cruz, CA) and β-actin: sc-47778 (Santa Cruz Biotechnology, Santa Cruz, CA). Secondary antibodies used included mouse anti-rabbit IgG-HRP:sc-2357(Santa Cruz Biotechnology, Santa Cruz, CA) and anti-mouse IgG (m-IgGκ BP-HRP):sc-516102 (Santa Cruz Biotechnology, Santa Cruz, CA). Protein–antibody complexes were made visible using enhanced chemiluminescence (ECL, Bonus, Amersham, Little Chalfont, UK) with the program IMAGE READER LAS-4000 in a LAS-4000 imaging system. Quantity One program were used to analyse the intensity of the signal. The values of each band were normalised with its β-actin, and were relative to the control sample to which the value 1 was assigned.

#### Statistical analysis

Data are shown as the mean ± standard deviation. Two-tailed Student’s t-test was used to analyse differences between groups. *p* < 0.05 was accepted as the statistical significance level.

### Molecular modelling

#### Protein preparation and ligand docking

The 3D structure of the tyrosine kinase domain of the HER2 was retrieved from the protein databank (PDB code 3RCD)^18^, and prepared for docking simulations with the protein preparation wizard tool from the Schrödinger suite^19^. This stage includes the modelling of missing loop regions (757–760, 867–883 and 992–999 and 1013–1015, mostly resolved in the alternative structure 3PP0) with Prime, addition of protons and evaluation of the protonation state of ionisable residues with Epik. A docking grid was defined on the basis of the co-crystallised pyrrolo[3,2-d]pyrimidine inhibitor^18^ with Glide^20^. After failed attempts to obtain reliable docking poses with the default flexible ligand docking procedure within Glide, an alternative strategy was conceived to enhance the conformational sampling, based on a 2-stepwise protocol: (i) the ligand bozepinib was built in a reliable 3D conformation within maestro, in independent *R* and *S* stereoisomeric configurations. (ii) each stereoisomer was subjected to an exhaustive conformational search using the Conformational Search (default options) algorithm within Macromodel^19^, generating 76 conformers for each stereoisomer. iii) each conformer was subjected to rigid-docking with Glide-XP^20^. Three poses for each stereoisomer were retained for further analysis, based on a double criteria combining the highest docking scores and the best shape and electrostatic superposition with the co-crystallised ligand pyrrolo[3,2-*d*]pyrimidine inhibitor and with the ATP analogue crystalised in the related EGFR kinase (ANP, PDB code 2EB3). Each of these binding modes for bozepinib in the HER2 structure was used as a starting point to manually dock all reported compounds within the series by means of the “Flexible Ligand Alignment" tool in Maestro.

#### Free energy perturbation calculations

The automated QligFEP protocol was used for free energy perturbation (FEP) calculations^21^. This protocol relies on molecular dynamics (MD) simulations obtained with the software Q^22^ under spherical boundary conditions, using A 25 Å sphere centred on the core of geometry of the ligand. Protein atoms in the boundary of the sphere had a positional restraint of 10 kcal/mol/Å^2^, while solvent atoms were subjected to polarisation and radial restrains using the surface constrained all-atom solvent (SCAAS)^21^^,^^23^ model to mimic the properties of bulk water at the sphere surface. Atoms lying outside the simulation sphere are tightly constrained (200 kcal/mol/Å^2^ force constant) and excluded from the calculation of non-bonded interactions. Long-range electrostatics interactions beyond a 10 Å cut off were treated with the local reaction field method^24^, except for the atoms undergoing the FEP transformation where no cut-off was applied. Solvent bond and angles were constrained using the SHAKE algorithm^25^. All titratable residues outside the sphere were neutralised and histidine residues were assigned a hydrogen atom on the ε nitrogen. Residue parameters were translated from the OPLS-AA/M force field^26^ and the parameters for the ligand and lipids were obtained with the FFLD software within Schrödinger suite^19^. The simulation sphere was warmed up from 0.1 to 298 K, during a first equilibration period of 0.61 nanoseconds, where an initial restraint of 25 kcal/mol/Å^2^ imposed on all heavy atoms was slowly released for all complexes. Thereafter the system was subjected to ten parallel replicates of unrestrained MD, where the FEP protocol is applied for each ligand transformation. Each of these MD replicates starts with a 0.25 nanosecond unbiased equilibration period, with different initial velocities. Thereafter the FEP protocol follows, which consists of 21 FEP λ-windows, distributed using a sigmoidal function and consisting of 10 ps each for every investigated ligand pair. To fulfil a thermodynamic cycle and calculate relative binding free energies, parallel FEP transformations are run in a sphere of water for each ligand pair. In these water simulations, the same parameters apply (i.e. sphere size, simulation time, etc.), and the relative binding free energy difference was estimated by solving the thermodynamic cycle utilising the Bennett acceptance ratio (BAR)^27^.

## Results and discussion

### Chemistry

**Scheme 1.**
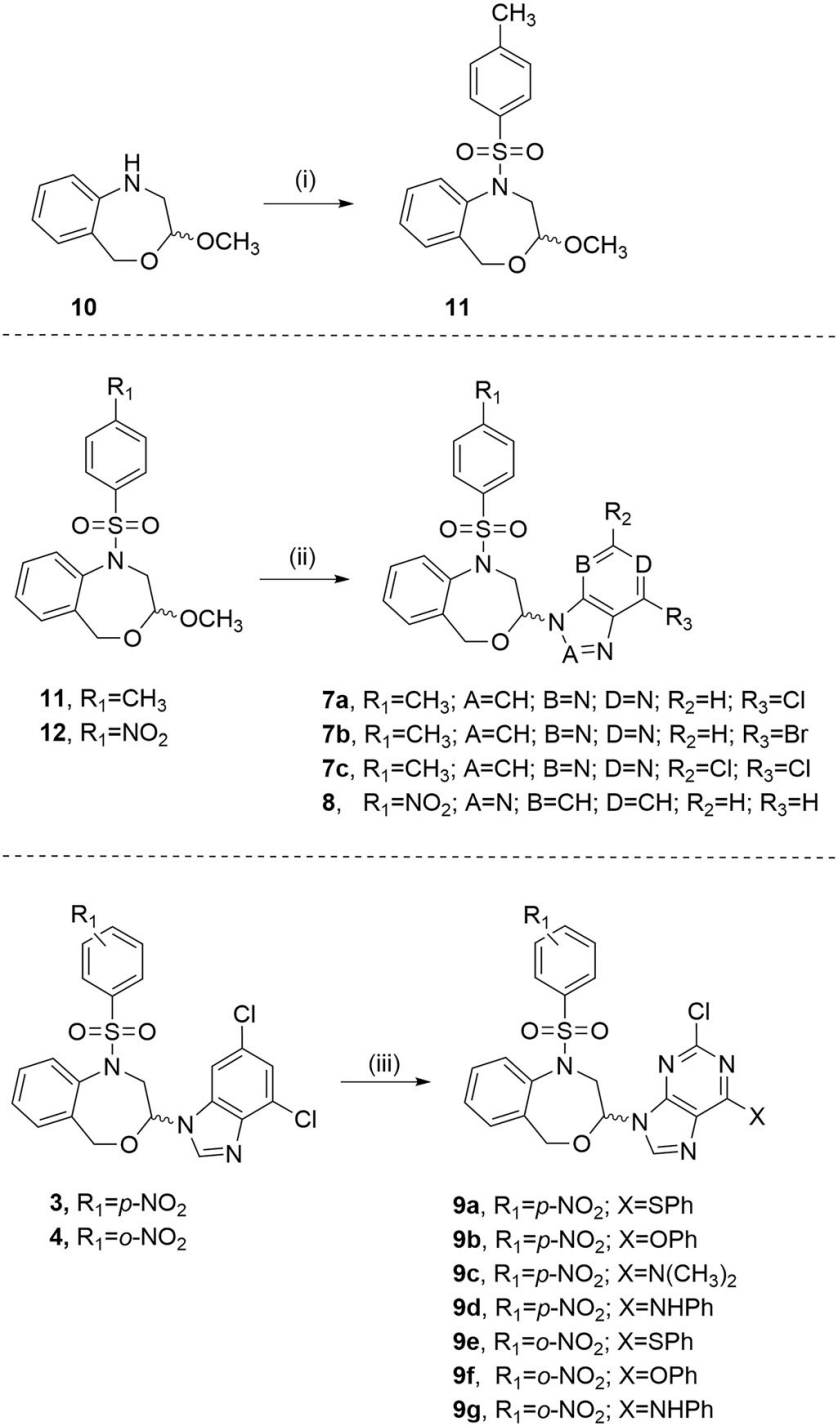
Reagents and conditions: (i) *p*-CH_3_PhSO_2_Cl, 0 °C, anhydrous CH_2_Cl_2_, argon, Method a: TEA, 110 °C microwave irradiation, 30 min, Method b: pyridine, 3 days, rt; (ii) purine or benzotriazole, TMSCl, HMDS, SnCl_4_ (1 M solution in CH_2_Cl_2_), 160 °C microwave irradiation, 5 min; (iii) PhSH, DMF, K_2_CO_3_, 1 h, rt for **9a** and **9e**, PhOH or PhNH_2_, DMF, TEA, 80 °C, 3 h for **9d** and **9g**, 6 h for **9f**, 20 h for **9b** and **9c**.

The target compounds (**7a**–**7c**, **8** and **9a**–**9g**) were synthesised as described in Scheme 1. Compound **11** was obtained from the *N*-unsubstituted acetal **10**^17^ by applying two different methods (i, Scheme 1). The best conditions were room temperature for 3 days using pyridine as a base. Although the use of microwave considerably reduces the reaction time, the yield is lower.

The preparation of **7a–7c** and **8** was achieved by the microwave-assisted Vorbrüggen one-pot condensation of the acetals **11** and **12**^16^ and the commercially available 6-chloro-, 6-bromo- and 2,6-dichloro-purines or benzotriazole using chlorotrimethylsilane (TMSCl), 1,1,1,3,3,3-hexamethyldisilazane (HMDS) and tin(IV) chloride as the Lewis acid in anhydrous acetonitrile. The reaction mixture was microwave-irradiated at a temperature of 160 °C for 5 min (ii, Scheme 1).

The nucleophilic substitution of the chlorine in position 6 of **3**^10^ and **4**^10^ by phenylthio (**9a** and **9e**), phenoxy (**9b** and **9f**), or phenylamine (**9d** and **9g**) was carried out in DMF using K_2_CO_3_ or TEA and applying different reaction conditions (see Experimental Part for details). Compound **9c** was obtained along with **9b**. The increase of the reaction time up to 20 h decomposes the DMF and the resulting dimethylamine competes as nucleophile as previously reported^28^.

### HER2 inhibition studies

Human epidermal growth factor receptor 2 (HER2) is overexpressed in about 20% of all breast cancers^29^. Bozepinib has proven to be a selective inhibitor of HER2 as we previously demonstrated by a kinase assay and then verified by immunoblot analysis in the HER2 positive SKBR-3 breast cancer cell line^12^. This prompted us to evaluated compounds **7**–**9** for their *in vitro* HER2 inhibitory effect. The IC_50_ profile of the target compounds was determined against isolated HER2 using a radiometric assay (Table 1).

**Table 1.** HER2 inhibition effect for compounds 3 and 7–9.

Compound	IC_50_ HER2 (µM)	Compound	IC_50_ HER2 (µM)
3	27.9	9b	13.3
7a	>100	9c	14.4
7b	>100	9d	55.5
7c	>100	9e	11.5
8	22.2	9f	24.6
9a	7.31	9g	>100

The nitro is crucial for the inhibition of the enzyme. Compounds that incorporate this group show IC_50_ ≤ 50 µM with the only exception of **9g**.

The replacement of the 2,6-dichloropurine by the benzotriazole moiety does not modify the inhibitory activity of the structures (**3**, IC_50_ HER2 = 27.9 µM versus **8**, IC_50_ HER2 = 22.2 µM).

The substitution of the chlorine at position 6 of the purine by the bulky groups phenylthio (**9a** and **9e**, IC_50_ 7.31 and 11.5 µM respectively) and phenyloxy (**9b** and **9f**, IC_50_ 13.3 and 24.6 µM respectively) favours the inhibition of the enzyme in contrast to the phenylamine substitution that leads to a decrease of the inhibition potency (**9d** and **9g**, IC_50_ 55.5 and > 100 µM, respectively). Compound **9c** that presents the dimethylamine substituent displays higher inhibition against HER2 than their corresponding phenylamine counterparts **9d** and **9g**.

Finally, the *p*-nitro substitution in the structures leads to higher HER2 inhibition compared with the corresponding *o*-nitro derivatives (IC_50_ **9a** < **9e**, **9b** < **9f** and **9d** < **9g**). In general, the *p*-nitro derivatives are twofold more active than the *o*-isomers.

The most potent compound against isolated HER2 is **9a** with an IC_50_ of 7.31 µM. This structure bears a *p*-nitrobenzenesulphonyl moiety and the phenylthio substituent in the purine fragment.

### Computational studies

To explore the SAR of the new series on HER2, we conducted a structure-based strategy as follows: (i) an exhaustive docking of the parent compound **3** on HER2 determined the most probable binding modes of this scaffold; (ii) the binding modes determined was used as a basis for parallel free energy perturbation (FEP) simulations, comparing the relative affinities of this compound with five selected ligands (**7a**–**7c**, **9a** and **9c**), representing different substitutions with varied effects on experimental binding affinity. The binding mode of **3** on HER2 was explored taking advantage of the crystallised tyrosine-kinase domain of the enzyme in complex with an ATP-competitive inhibitor (PDB code 3RCD^18^). The selected structure presented a more open P-loop, as compared to the alternative structure 3PP0, allowing a better fitting of **3** (docking studies on that structure did not yield any reliable binding mode, data not shown). After independent docking conducted on the *R* and *S* stereoisomers of **3**, three poses for each stereoisomer were retained for further analysis. We have previously demonstrated that both enantiomers are equally potent^10^. Each of these occupied the ATP binding site, overlaying the ANP analogue co-crystalised in the related EGFR kinase, as well as the unrelated pyrrolo[3,2-*d*]pyrimidine co-crystallised with HER2 (see Methods). The six binding poses were the starting point of independent series of free energy perturbation (FEP) calculations aimed to explain the observed SAR within the series of bozepinib analogues (Table 2).

**Table 2.** Calculated and experimental free energy binding differences between selected derivatives and bozepinib (3) using the binding modes illustrated in Figure 2.

Perturbation (3 -> L) ligand (L)	ΔΔG (kcal/mol)			
	Experiment^a^	Calculated		
		*R* isomer	*S* isomer	Racemic
7a	>0.76	2.51 ± 0.21	1.19 ± 0.23	1.85 ± 0.31
7b	>0.76	1.98 ± 0.28	1.66 ± 0.25	1.82 ± 0.37
7c	>0.76	1.84 ± 0.21	0.31 ± 0.11	1.08 ± 0.23
9a	−0.8	−3.27 ± 0.63	−1.52 ± 0.86	−2.40 ± 1.05
9c	−0.44	−2.38 ± 0.33	−2.99 ± 0.29	−2.69 ± 0.44
9d	0.41	−2.17 ± 1.00	0.27 ± 0.22	−0.95 ± 1.03

^a^Experimental binding free energy differences extracted from the corresponding IC_50_ values using the relationship ($\Delta \Delta G_{\mathrm{bind},\;\;\text{exp}\;}^{0}=\mathrm{RTln}(\frac{{IC}_{50}(3)}{{IC}_{50}(L)})$).

**Figure 2.**
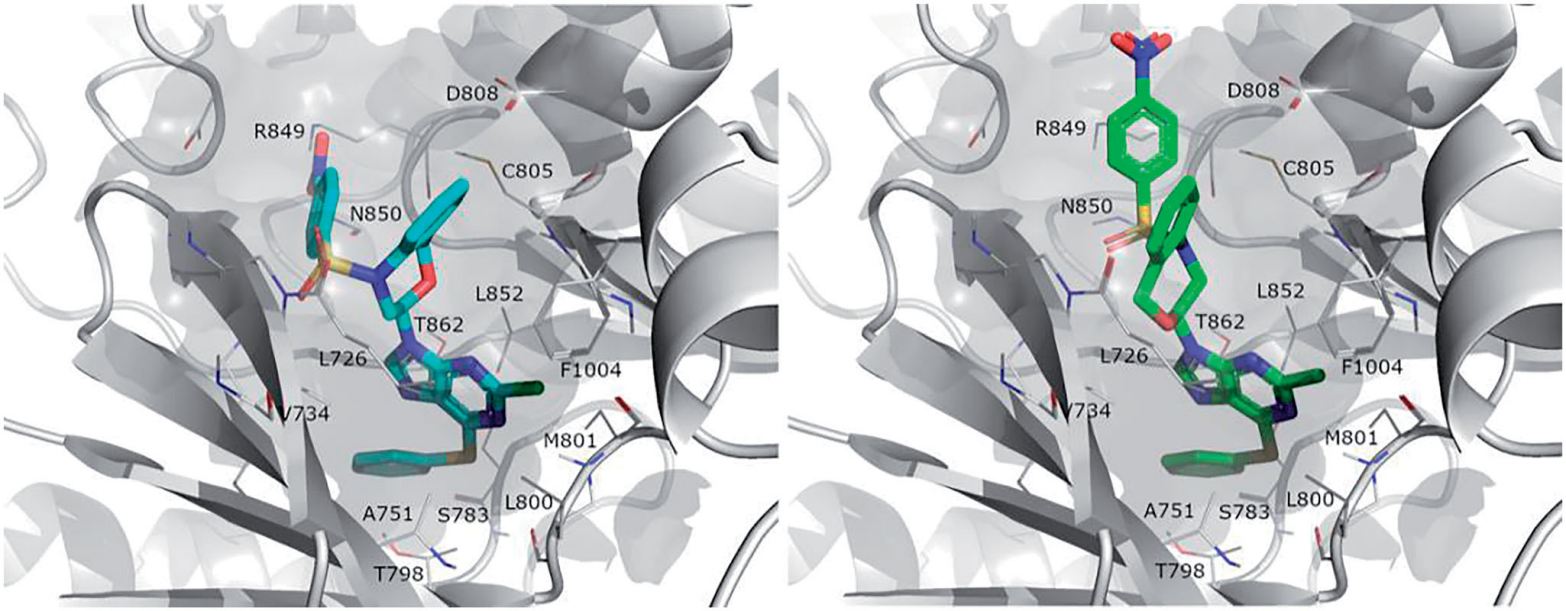
Binding mode of compound **9a** obtained by docking. Both *R* (left, blue) and *S* isomers (right, green) were docked independently, and used as a basis for parallel FEP simulations.

In each case, we evaluated the pairwise binding free energy difference between bozepinib and five analogues, exploring different structural features: (i) the detrimental effect or removing the nitro group, with independence of the position of the halogen(s) (**7a**, **7b, 7c**). (ii) The increase in affinity as a result of replacing one halogen by a phenylthio group (**9a**) or a dimethylamino group (**9c**). From the six docking alternatives filtered in the docking stage, only one pose *per* stereoisomer resulted in a qualitative correlation with the observed SAR of these compounds, the data shown in Table 2. Noteworthy, the orientation of the purine ring is equivalent between the two isomers, while the rest of the molecule is still bound in a similar area within the binding site, as shown in Figure 2. Hence, the purine ring matches the position of the purine on ANP in EGFR, with the chlorine group in position 6 pointing towards the most buried region of the binding site (overlaying to the exocyclic amino group of ATP/ANP). This is precisely the position that can be replaced by either dimethylamino (**9c**) or a phenylthio group (**9a**), in the last case occupying the region that was accommodating the aminoaryl substituent of the co-crystallised pyrrolo[3,2-*d*]pyrimidine within residues Ala751, Ser783, Thr798 and Leu852 (see Figure 2).

Our FEP calculations show indeed an enhancement of affinity by these two substitutions, in agreement with the experimental data (Table 1). Conversely, the replacement of the O or S atoms in **9a** and **9c**, respectively, by an NH functionality (**9d**) seems to be unfavourable for the binding affinity (Table 1). The corresponding FEP simulation of this compound is actually non-conclusive, since the results for either stereoisomer are contradictory (i.e. only the *S*-isomer is found to bind with less affinity than the reference compound), and the resulting small increase in binding affinity predicted for the racemic mixture is within the error bars of the calculation (−0.95 ± 1.03 kcal/mol, see Table 2).

Along the compounds studied by molecular modelling, we consistently observed that the region occupied by the nitro group is somehow different between the *R* and *S* stereoisomers. In both cases, however, it is facilitated by the more open conformation of the P-loop observed as an induced-fit effect in the structure selected for docking and MD purposes^18^. The β-hairpin encompassing this loop would accommodate the sulphonamide group, through mainchain or sidechain (Lys753) interactions. Though partially solvent exposed, the *p*-nitro group is placed between Asp808 and Arg849, offering a plausible justification for its key role in the affinity of this series. On the basis of these results, where both the *R* and *S* isomers show a similar orientation and predicted SAR in line with the experimental data, we postulate that the ATP binding site of the HER2 shows tolerance for the stereogenic centre, in other words that the binding of these compounds would not be stereoselective. Table 2 shows an estimation of the relative free energy shift for each compound, as compared to bozepinib, for the racemic mixture estimated as an arithmetic average between the values independently obtained from the *R* and *S* isomers. We have previously shown no significant difference in activity of bozepinib enantiomers in MCF-7 and MDA-MB-231 tumour cells^10^.

### Biological studies

We then investigated the effects of the target compounds on cell proliferation on the human breast cancer cell lines MCF-7 and SKBR-3, the human colon carcinoma cell line HCT-116 and the human malignant melanoma cell line A-375. The antiproliferative activity of **7**–**9** was evaluated using sulphorhodamine-B colorimetric assay after 72 h of treatment (Table 3).

**Table 3.** Antiproliferative activities for compounds **3** and **7–9** against the cancerous cell lines MCF-7, SKBR3, HCT-116 and A-375.

Compound	IC_50_ MCF-7 (µM)	IC_50_ SKBR3 (µM)	IC_50_ HCT-116 (µM)	IC_50_ A375 (µM)
**3**	0.35 ± 0.01^10^	0.33 ± 0.00^12^	0.57 ± 0.02^10^	1.05 ± 0.01
**7a**	0.12 ± 0.00	1.08 ± 0.02	1.03 ± 0.05	0.78 ± 0.03
**7b**	9.57 ± 0.03	2.30 ± 0.04	3.10 ± 0.01	3.08 ± 0.01
**7c**	0.86 ± 0.06	0.42 ± 0.01	0.86 ± 0.03	0.78 ± 0.05
**8**	8.00 ± 0.02	5.22 ± 0.05	6.56 ± 0.04	20.17 ± 0.15
**9a**	26.46 ± 0.03	71.69 ± 0.50	19.62 ± 0.01	13.30 ± 0.07
**9b**	22.40 ± 0.03	>100	>100	>100
**9c**	20.70 ± 0.02	41.66 ± 0.79	87.00 ± 0.19	>100
**9d**	13.80 ± 0.02	>100	>100	>100
**9e**	20.50 ± 0.06	>100	>100	92.23 ± 8.90
**9f**	18.00 ± 0.02	>100	>100	>100
**9g**	22.50 ± 0.02	>100	>100	>100

Compounds that bear halogen-substituted purines (**7a** and **7c**) show IC_50_ values ≤ 1 μM against the four cell lines, with the exception of compound **7b** with IC_50_ values ranging from 2.30 µM to 9.57 µM. The similarity of these compounds in terms of antiproliferative effects did not allow us to determine a clear structure–activity relationship.

All experiments were conducted in duplicate and gave similar results. The data are mean ± SD of three independent determinations. MCF-7: human breast cancer cell line; SKBR3: human breast cancer cell line; HCT-116: human colon cancer cell line and A-375: human melanoma cancer cell line.

The nitro and methyl groups, in general maintain the biological effect against the four cell lines (**7a**, **7b** and **7c** versus **3**). The 6-chloro (**7a**) and 2,6-dichloropurine (**7c**) derivatives show IC_50_ values in the sub-micromolar range.

The substitution of the purine by benzotriazole considerable decreases the antiproliferative effect against MCF-7, SKBR3 and HCT-116 (**8** versus **3**).

Surprisingly, our results show that the substitution of the chlorine atom at position 6 of the purine by the bulky groups phenylthio (**9a** and **9e**), phenoxy (**9b** and **9f**), or phenylamine (**9d** and **9g**) considerably decreases the antiproliferative effect. This effect is less pronounced in MCF-7 cells with IC_50_ values ranging from 13.80 µM (**9d**) to 26.46 µM (**9a**). In comparing the cytotoxicity of this series of compounds, **9a** is the most potent structure against SKBR3, HCT-116 and A375.

Compound **7c** displays the best antiproliferative effect against all the tumour cell lines studied (IC_50_ 0.42–0.86 µM).

We selected **7c** to decipher the mechanism by which it induces cell death. For this purpose, we studied HER2, cytochrome c, bax and caspase 3.

**Figure 3.**
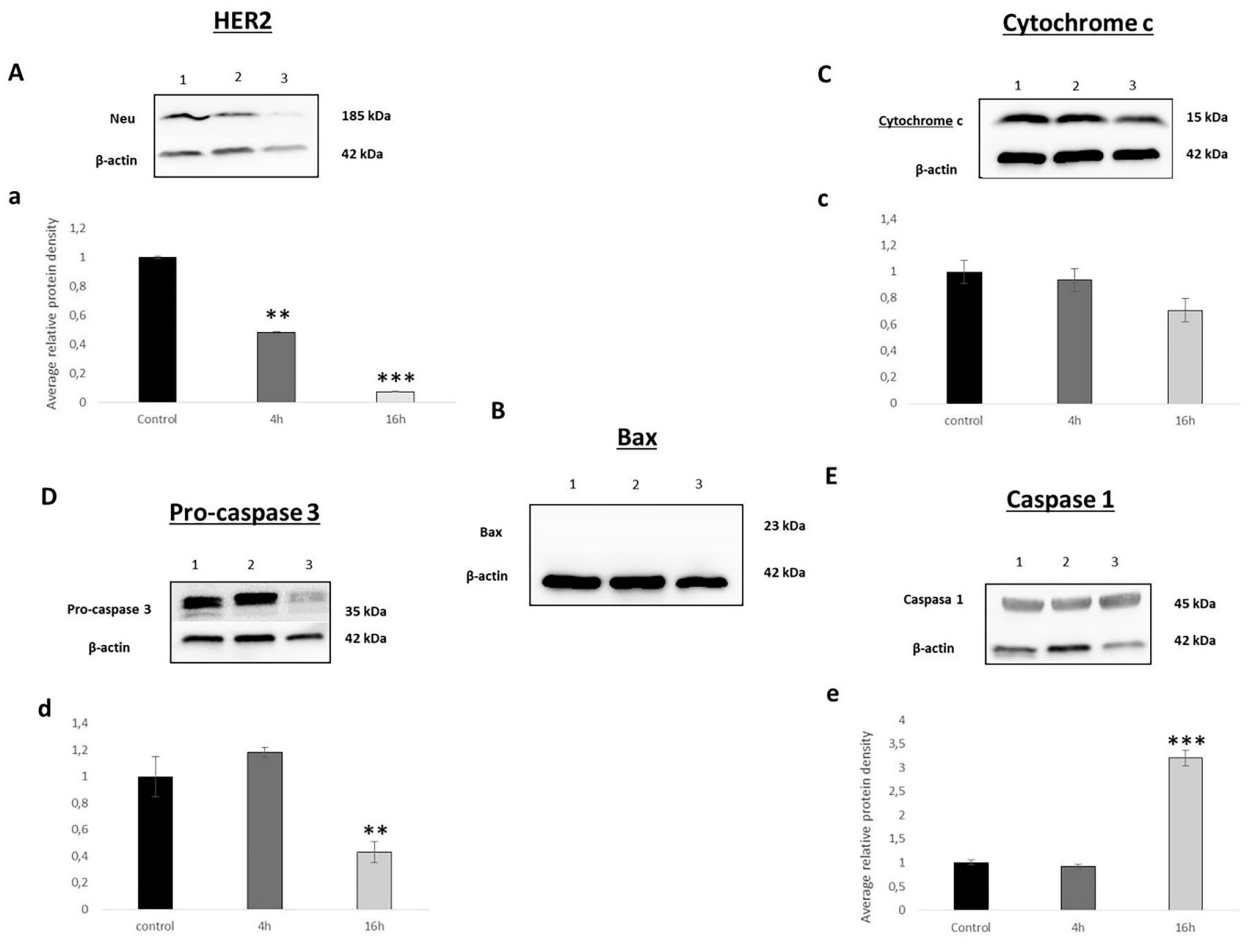
Western blot analysis of (A) HER2, (B) Bax, (C) cytochrome c, (D) pro-caspase 3, (E) caspase 1. Band 1. Control, band 2. after 4 h treatment, band 3. after 16 h treatment. Relative quantification of the western blot proteins normalised with β-actin signal and relative to mock-treated cells (value 1) (a, b, c and d). Data expressed as a mean ± SD from three independent experiments performed in triplicated (*p < 0.05 versus control, **p < 0.01 versus control and ***p < 0.001 versus control).

After normalisation and comparison to the relative value of the control, a clear decrease in the expression of the HER2 was observed reaching values of 0.48 and 0.07 after 4 h and 16 h of treatment respectively (Figure 3(A,a)). Since **7c** has not shown inhibition of isolated HER2 in the kinase assay (Table 1), these results suggest that, unlike bozepinib, **7c** does not act directly on the HER2 receptor but instead upstream. Trastuzumab, used currently as first-line clinical treatment for metastatic HER2 positive breast tumours, has improved overall survival in patients, although there are still a considerable percentage of patients with metastatic disease that ultimately developed resistance to this drug^30^. Thus, **7c** may be used in the future as a possible strategy to overcome treatment resistance.

Bcl-2 family proteins play a key role in the mitochondrial cell death pathway by disrupting mitochondrial membrane potential and the release of apoptogenic factors, such as cytochrome c from mitochondria, in the cytosol. Bax is a member of the Bcl‐2 family and core regulators of the intrinsic pathway of apoptosis. Our results demonstrate none expression of Bax neither in the control nor in the treated cells (Figure 3(B,b)). These results were in accordance with what was obtained by analysing cytochrome c. As shown in Figure 3(C,c), the normalised values were 0.93 and 0.70 after 4 h and 16 h of treatment. **7c** has not modified the expression of cytochrome c, an electron acceptor protein located in the mitochondrial intermembrane and identified as a key molecule in intrinsic pathway of apoptosis indicating that there is no activation of the intrinsic apoptotic pathway mediated by mitochondrial effects^31^^,^^32^

Compared with other members of the caspases family, caspase-3 is at the end of the caspase cascade and is activated by both the intrinsic and extrinsic death pathways in apoptosis^32^^,^^33^. In our analysis, and at our surprise, we have not observed any modification statistically significant in the expression of pro-caspase 3 at 4 h, while at 16 h this expression was approximately halved. Thus, the band corresponding to 4 h and 16 h after treatment had a value of 1.18 and 0.43, respectively (Figure 3(D,d)). This result indicates that cell death induced by **7c** is not performed via apoptosis as we supposed *a priori*.

In recent years, researchers began to conduct in-depth investigations on cell death modes other than apoptosis, and the newly discovered pyroptosis gradually came into the field. Different studies have proved that pyroptosis can also shrink tumours and inhibit cells proliferation^34^^,^^35^. Traditionally pyroptosis is defined as caspase-1-mediated cell death, for that we studied the effect of **7c** treatment on SKBR cell line. Our results demonstrated an increase in caspase 1 after 16 h of **7c** exposition with normalised values reaching 3.1 (Figure 3(E,e)). Furthermore, recent study demonstrated that the apoptotic effector caspase, caspase-3 is capable of triggering pyroptosis^36^^,^^37^. In fact, Rogers et al. demonstrate that the decrease of pro-caspase-3 and increase of caspase 1 expression was related to the induction of secondary necrosis/pyroptosis^38^. This can explain the decrease of pro-caspase 3 expression observed after 16 h of **7c** exposition. For all the above, our finding suggests that **7c** unlike **3**, induces cell death via pyroptosis pathway.

## Conclusions

We have designed and synthesised a series of 1,5-dihydro-4,1-benzoxazepines. The inhibitory activity against isolated HER-2 and the antiproliferative effects of the target compounds against the cancerous cell lines MCF-7, SKBR3, HCT-116 and A-375 have been determined. Our results have demonstrated that the presence of the nitro group is essential to inhibit HER2 but not cytotoxicity, since the substitution of the nitro for the methyl group maintains the toxicity against cancer cells. **7c** shows the best activity against all the tumour cell lines studied (IC_50_ 0.42–0.86 µM). This compound does not act directly on the HER2 receptor but acts upstream. To elucidate the mechanism by which this compound induces cell death we have studied the expression of cytochrome c, bax and caspase 3. Although **7c** does not modified the expression of any of these key factors in the intrinsic pathway of apoptosis, an increase in caspase 1 levels was observed. These findings suggest that this compound induces cell death via pyroptosis. More studies should be carried out in order to delve deeper into this pathway and we plan to conduct *in vivo* studies in the near future.

## Supplementary Material

Supplemental Material
