# Supplementary material for: Design, synthesis, HER2 inhibition and anticancer evaluation of new substituted 1,5-dihydro-4,1-benzoxazepines
Source: J Enzyme Inhib Med Chem. 2021 Jul 12;36(1):1553–63. doi: 10.1080/14756366.2021.1948841 (PMC8279156; doi:10.1080/14756366.2021.1948841)
Supplement: Supplemental Material [file IENZ_A_1948841_SM6583.pdf]

Supplemental Material

Design, synthesis, HER2 inhibition and anticancer evaluation of new substituted 1,5-dihydro-4,1-benzoxazepines

Olga Cruz-López[1,2,*], Matilde Ner[1], Francho Nerín-Fonz[3], Yaiza Jimenez-Martínez[2,4,5], David Araripe[3], Juan A. Marchal[2,4,5]; Houria Boulaiz,[2,4,5] Hugo Gutiérrez-de-Terán[3], Joaquín M. Campos,[1,2] Ana Conejo-García[1,2,*]

[1] *Department of Medicinal and Organic Chemistry, Faculty of Pharmacy, University of Granada, Granada, Spain*

[2] *Biosanitary Institute of Granada (ibs.GRANADA), SAS-University of Granada, Granada, Spain.*

[3]*Department of Cell and Molecular Biology, Uppsala University, Uppsala SE-75124, Sweeden.*

[4]*Biopathology and Medicine Regenerative Institute, University of Granada, Granada, Spain.*

[5]*Excellence Research Unit "Modeling Nature" (MNat), Department of Human Anatomy and Embryology, University of Granada, Granada, Spain.*

* *Corresponding authors*

aconejo@ugr.es, olgacl@ugr.es *Department of Medicinal and Organic Chemistry, Faculty of Pharmacy, University of Granada, Campus Cartuja s/n, Granada 18071, Spain*

1. NMR Spectra **2-12**

2. Mass Spectra **13-18**

1. ^{1}H and ^{13}C NMR Spectra

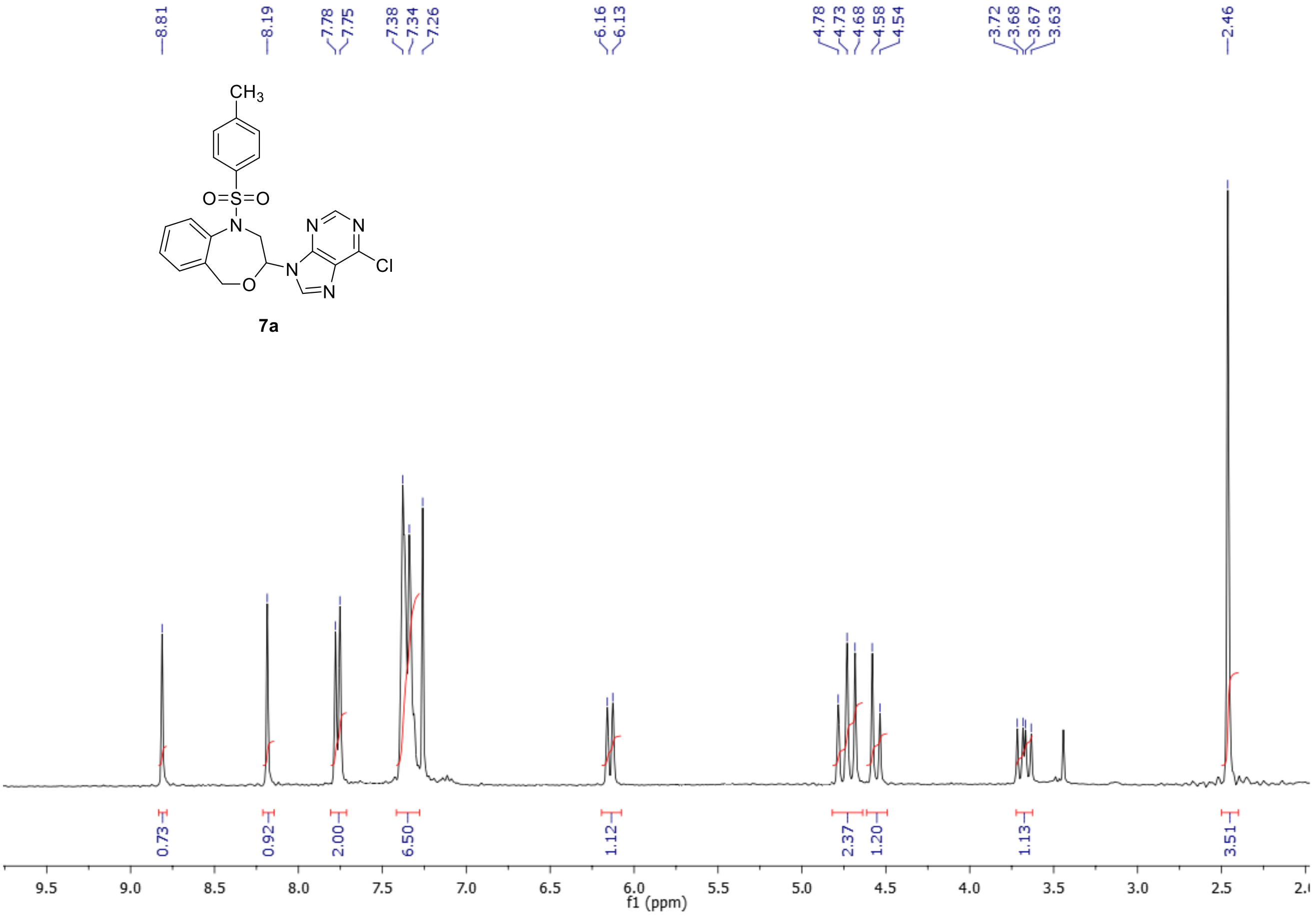

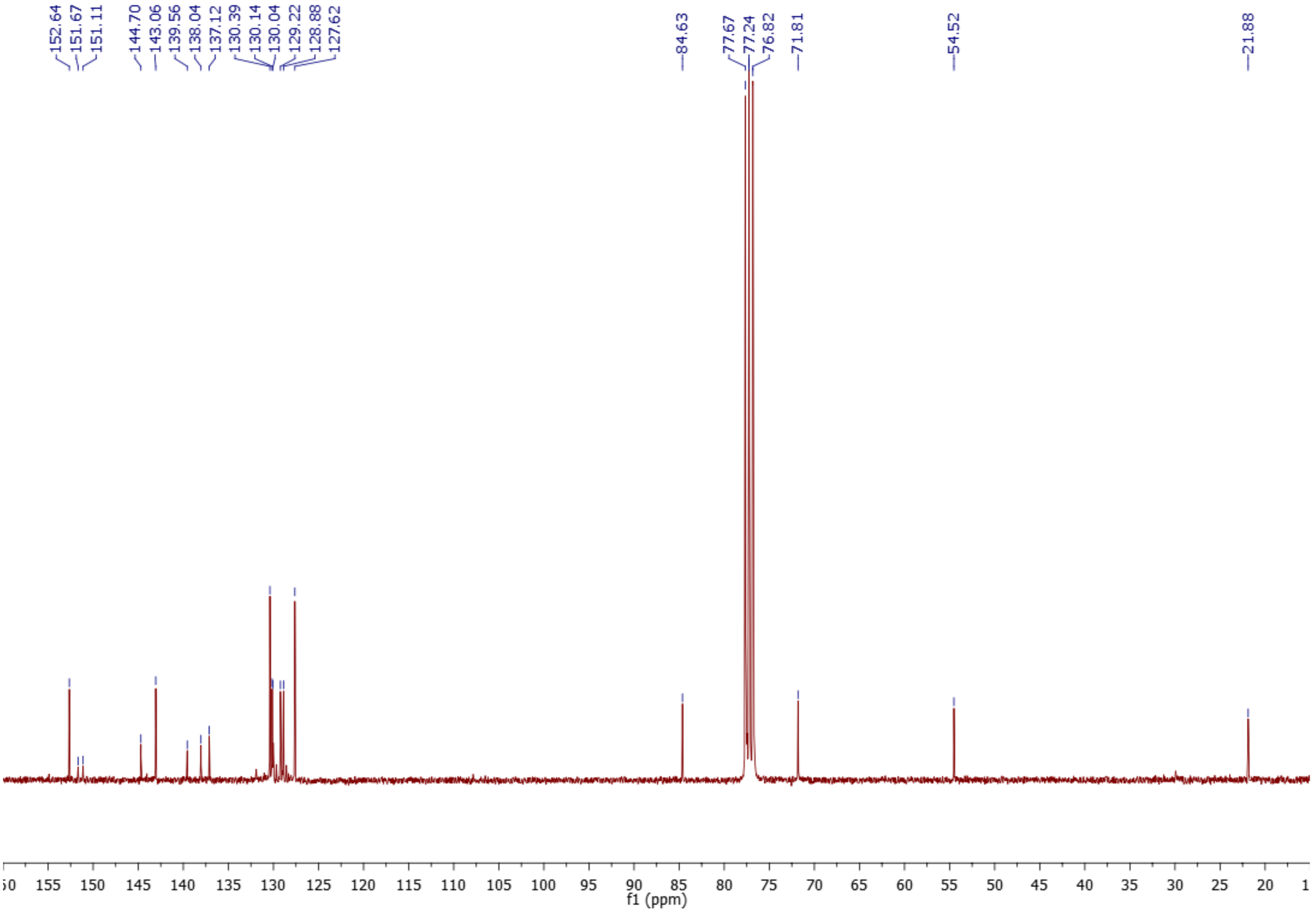

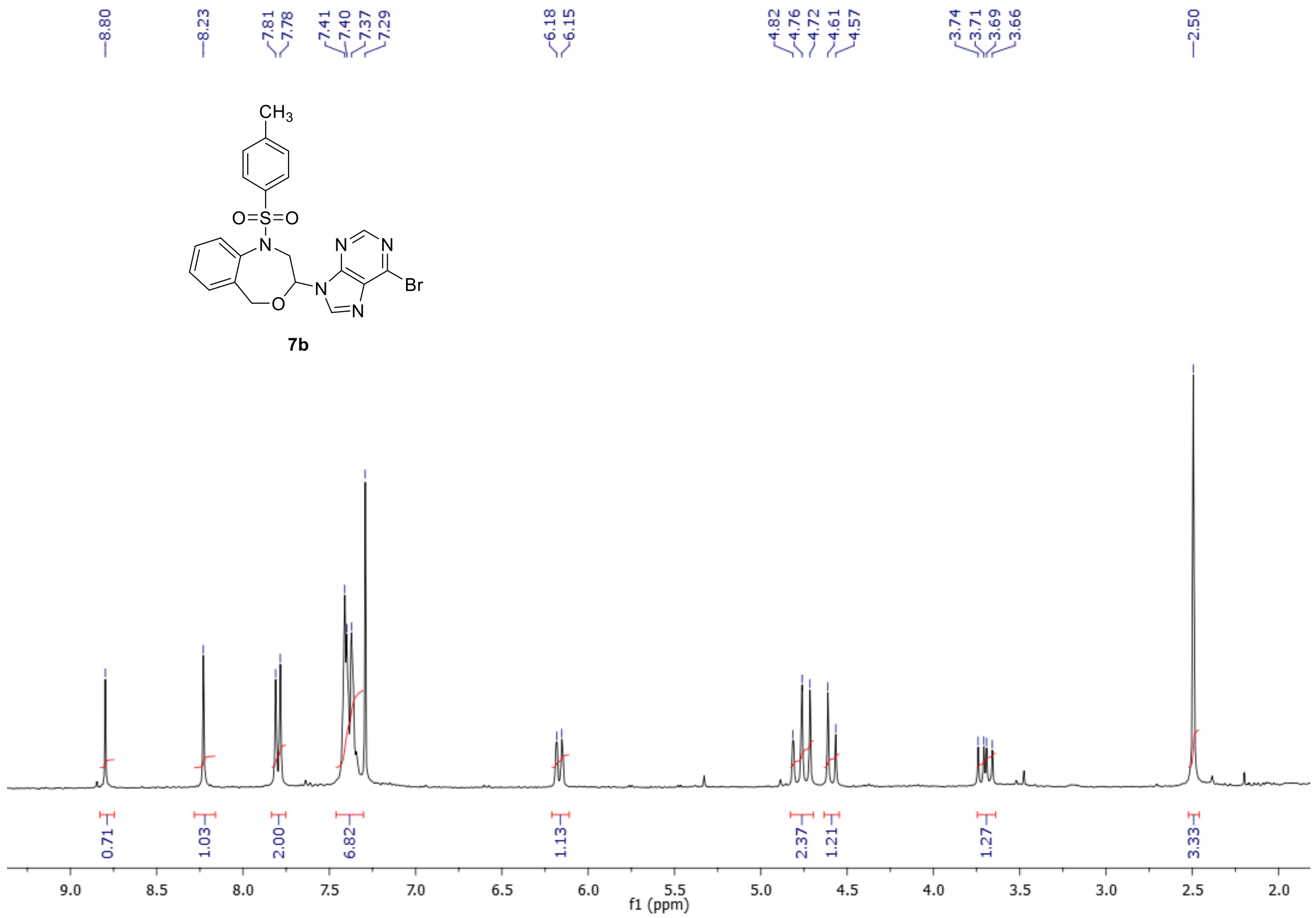
8.80
8.23
7.81
7.78
7.41
7.40
7.37
7.29
6.18
6.15
4.82
4.76
4.72
4.61
4.57
3.74
3.71
3.69
3.66
2.50
CH3
O=S=O
N
N
N
N
N
O
Br
7b
0.71
1.03
2.00
6.82
1.13
2.37
1.21
1.27
3.33
f1 (ppm)

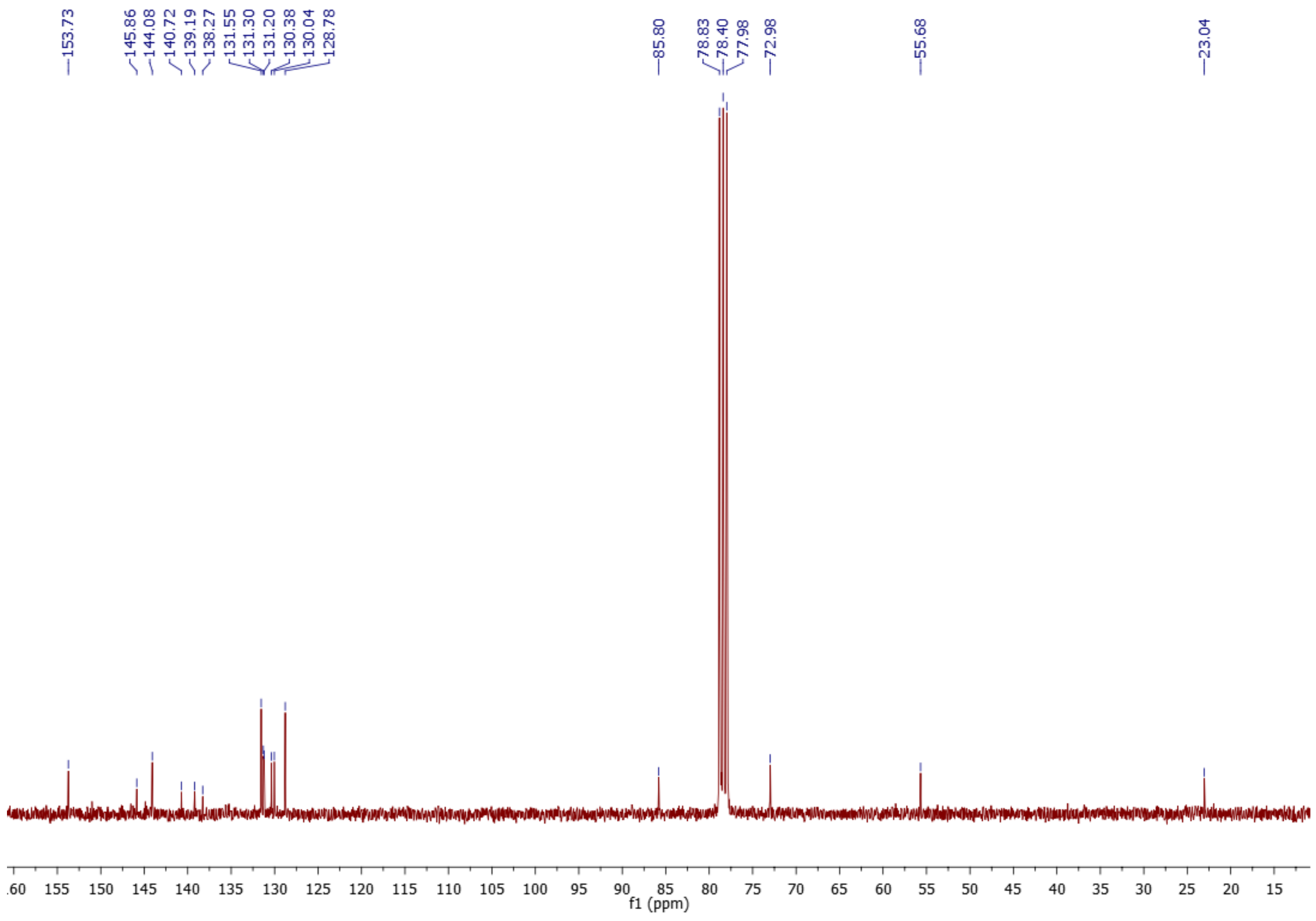
153.73
145.86
144.08
140.72
139.19
138.27
131.55
131.30
131.20
130.38
130.04
128.78
85.80
78.83
78.40
77.98
72.98
55.68
23.04
f1 (ppm)

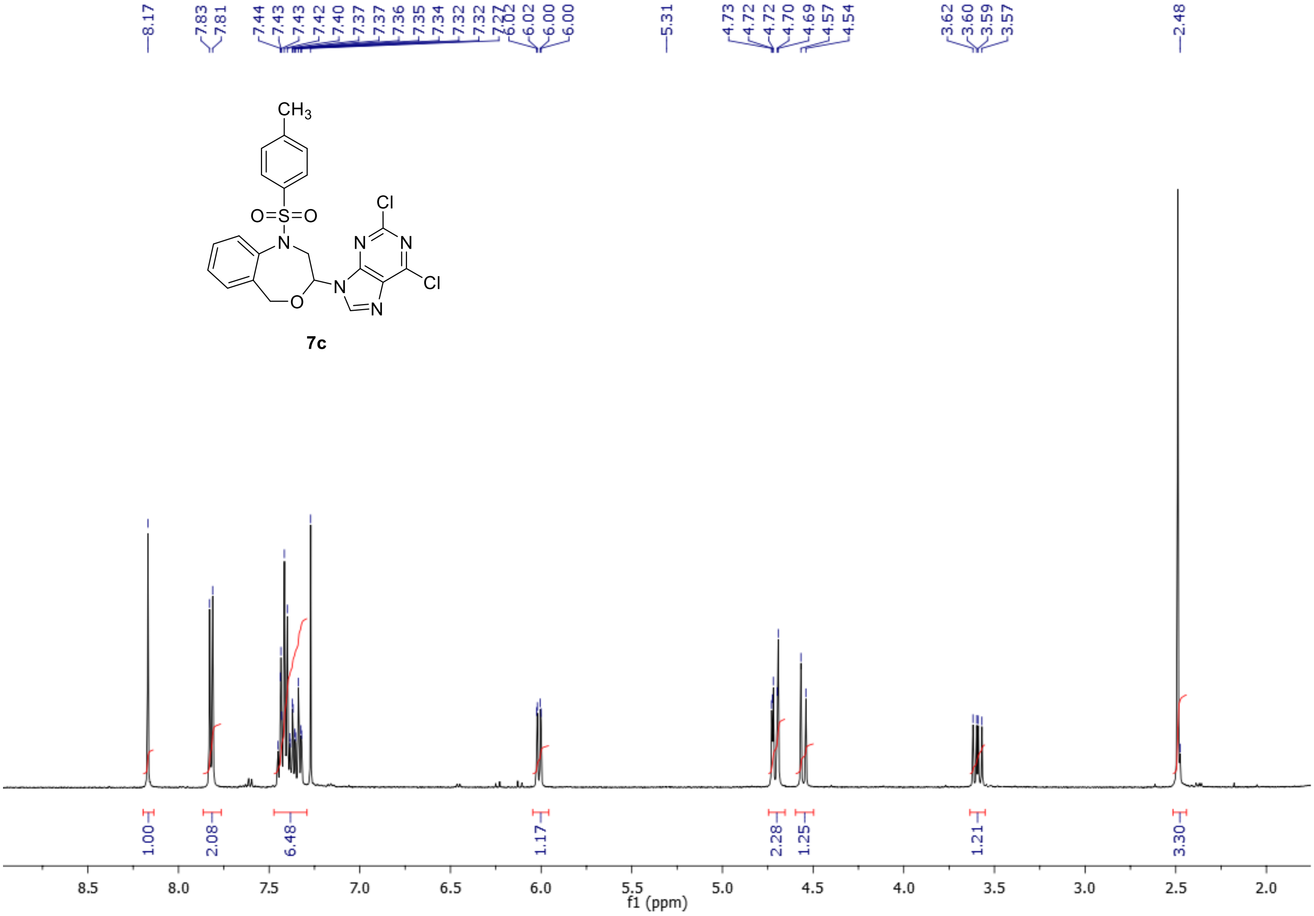
8.17
7.83
7.81
7.44
7.43
7.43
7.42
7.40
7.37
7.37
7.36
7.35
7.34
7.32
7.32
7.27
6.02
6.02
6.00
6.00
5.31
4.73
4.72
4.72
4.70
4.69
4.57
4.54
3.62
3.60
3.59
3.57
2.48
CH3
O=S=O
N
Cl
N
N
N
Cl
O
N
7c
1.00
2.08
6.48
1.17
2.28
1.25
1.21
3.30
f1 (ppm)

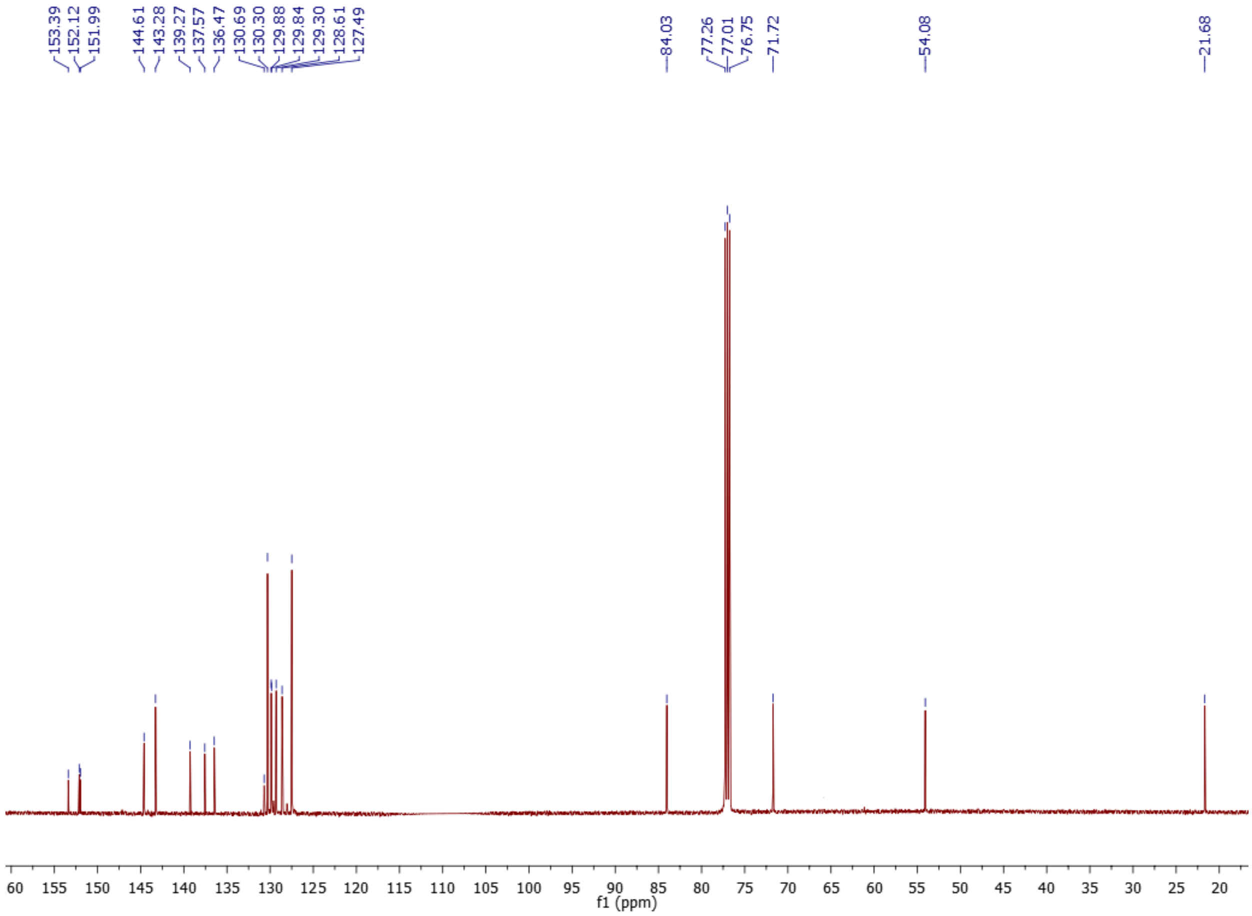
153.39
152.12
151.99
144.61
143.28
139.27
137.57
136.47
130.69
130.30
129.88
129.84
129.30
128.61
127.49
84.03
77.26
77.01
76.75
71.72
54.08
21.68
f1 (ppm)

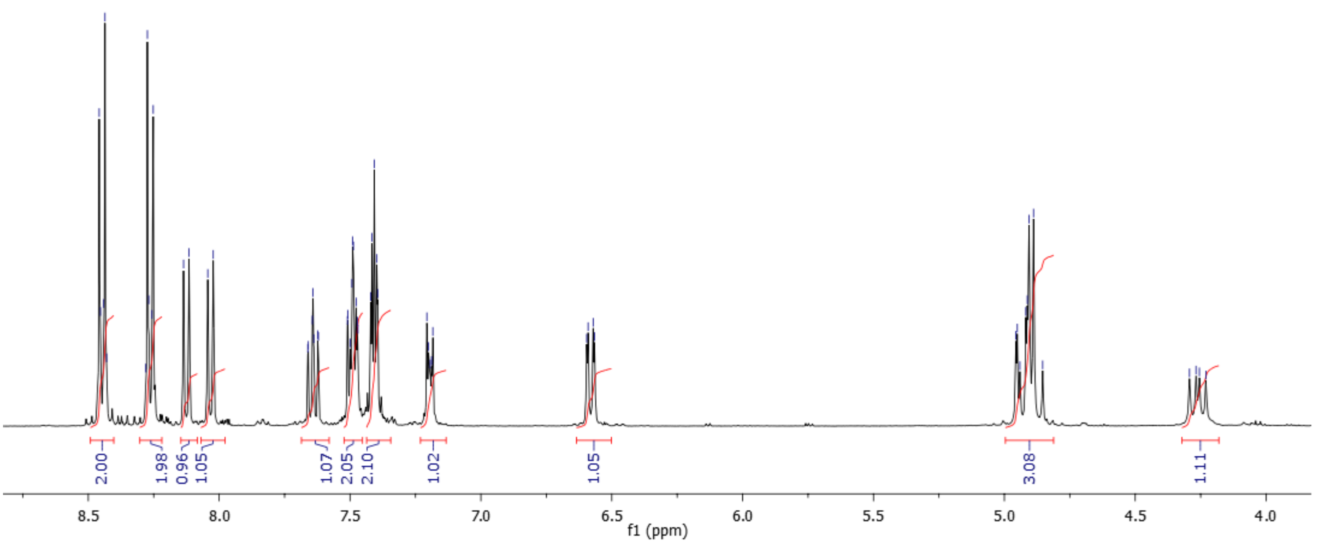
8.46
8.45
8.44
8.44
8.27
8.27
8.26
8.25
8.14
8.12
8.04
8.02
7.64
7.49
7.49
7.49
7.42
7.41
7.40
7.21
7.20
7.19
7.19
7.18
6.60
6.59
6.57
6.57
4.96
4.95
4.94
4.92
4.91
4.91
4.89
4.85
4.29
4.27
4.25
4.23
2.00
1.98
0.96
1.05
1.07
2.05
2.10
1.02
1.05
3.08
1.11
8.5
8.0
7.5
7.0
6.5
6.0
5.5
5.0
4.5
4.0
f1 (ppm)

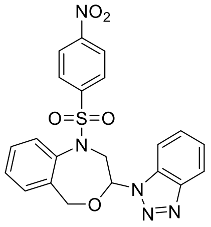
NO2
O=S=O
N
O
N
N=N
8

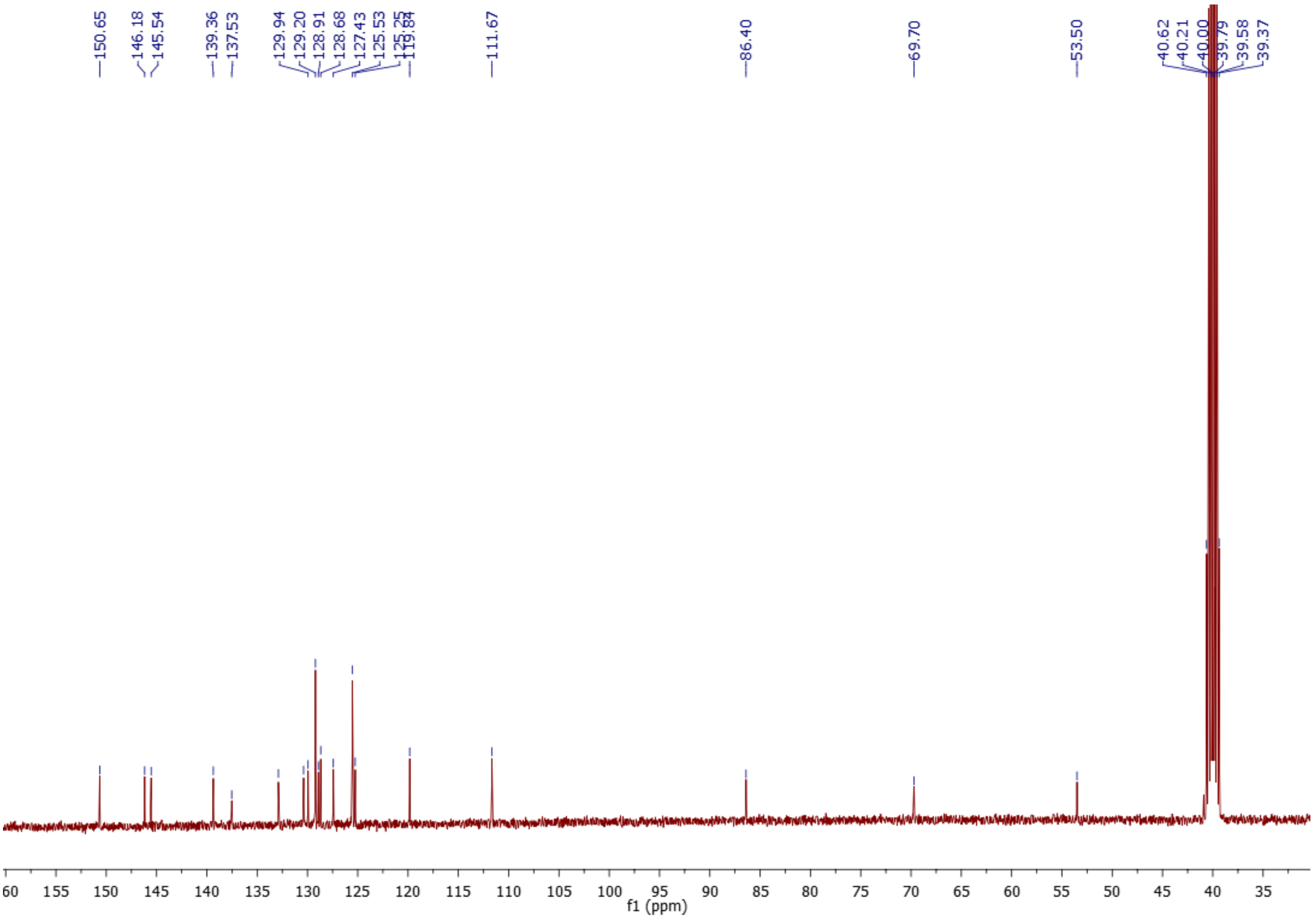
150.65
146.18
145.54
139.36
137.53
129.94
129.20
128.91
128.68
127.43
125.53
125.25
119.84
111.67
86.40
69.70
53.50
40.62
40.21
40.00
39.79
39.58
39.37
160
155
150
145
140
135
130
125
120
115
110
105
100
95
90
85
80
75
70
65
60
55
50
45
40
35
f1 (ppm)

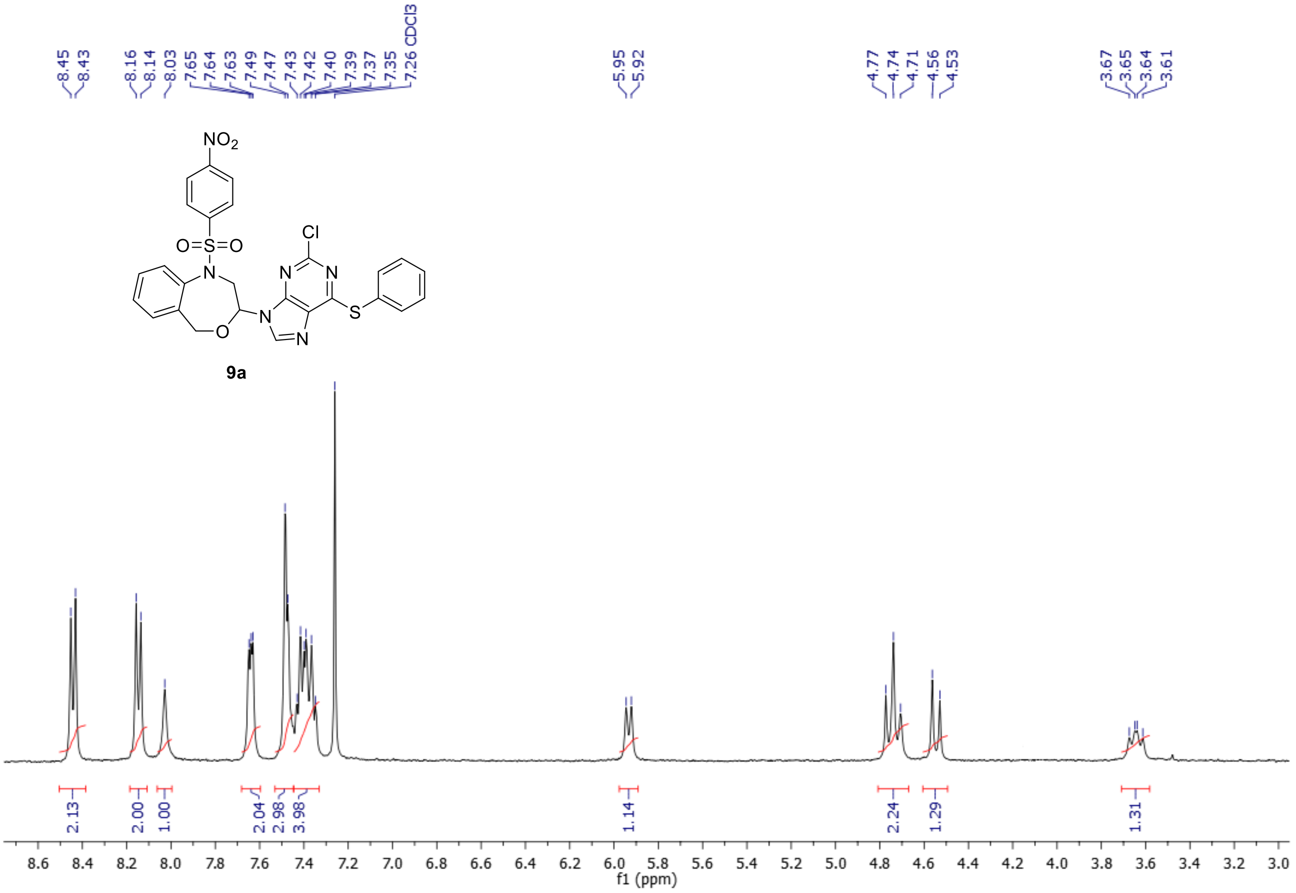
8.45
8.43
8.16
8.14
8.03
7.65
7.64
7.63
7.49
7.47
7.43
7.42
7.40
7.39
7.37
7.35
7.26 CDCl3
5.95
5.92
4.77
4.74
4.71
4.56
4.53
3.67
3.65
3.64
3.61
NO2
O=S=O
N
O
N
Cl
N
N
N
S
9a
2.13
2.00
1.00
2.04
2.98
3.98
1.14
2.24
1.29
1.31
f1 (ppm)

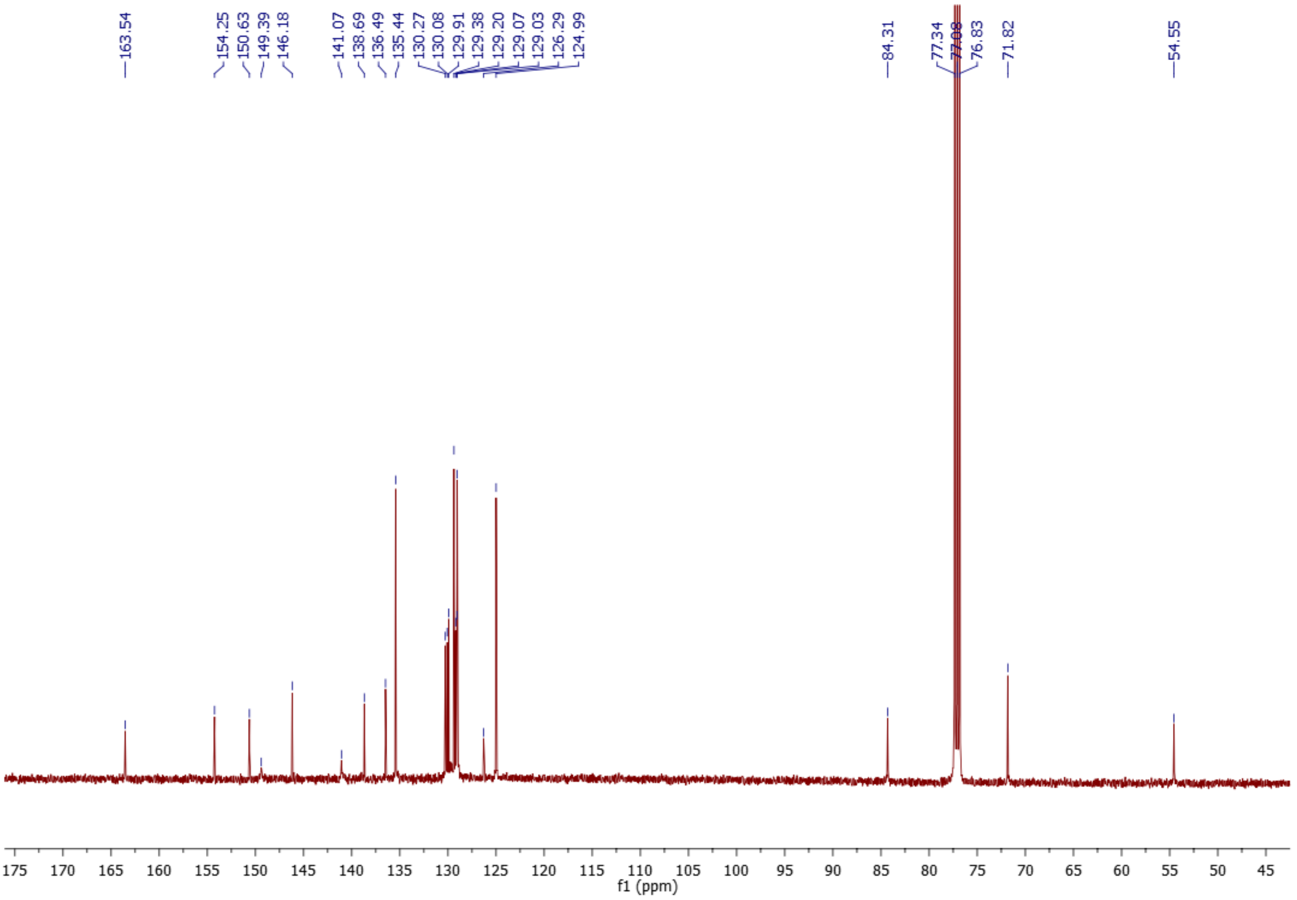
163.54
154.25
150.63
149.39
146.18
141.07
138.69
136.49
135.44
130.27
130.08
129.91
129.38
129.20
129.07
129.03
126.29
124.99
84.31
77.34
77.08
76.83
71.82
54.55
f1 (ppm)

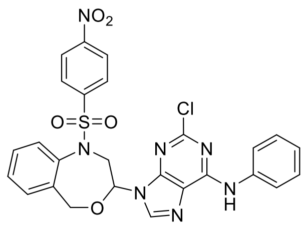
NO2
O=S=O
N
O
N
N
N
N
Cl
N
H

9d

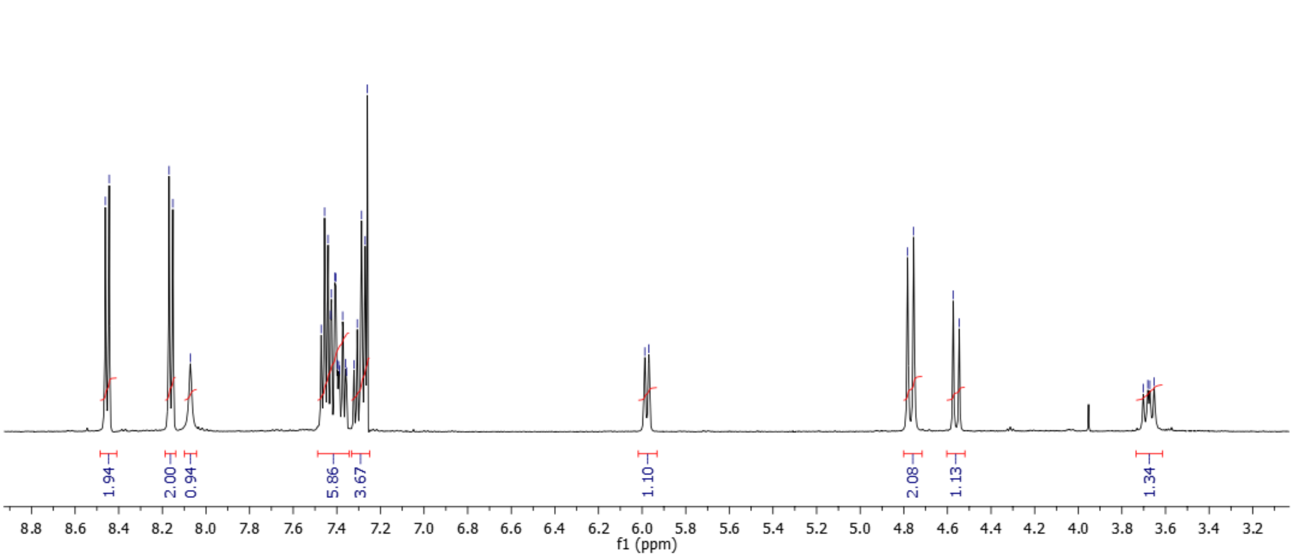
8.46
8.44
8.17
8.15
8.07
7.47
7.46
7.44
7.43
7.42
7.41
7.40
7.40
7.39
7.39
7.37
7.36
7.36
7.32
7.31
7.29
7.27
7.26
5.99
5.97
5.30
4.78
4.75
4.57
4.55
3.70
3.68
3.67
3.65
3.49
3.47
1.94
2.00
0.94
5.86
3.67
1.10
2.08
1.13
1.34
8.8 8.6 8.4 8.2 8.0 7.8 7.6 7.4 7.2 7.0 6.8 6.6 6.4 6.2 6.0 5.8 5.6 5.4 5.2 5.0 4.8 4.6 4.4 4.2 4.0 3.8 3.6 3.4 3.2
f1 (ppm)

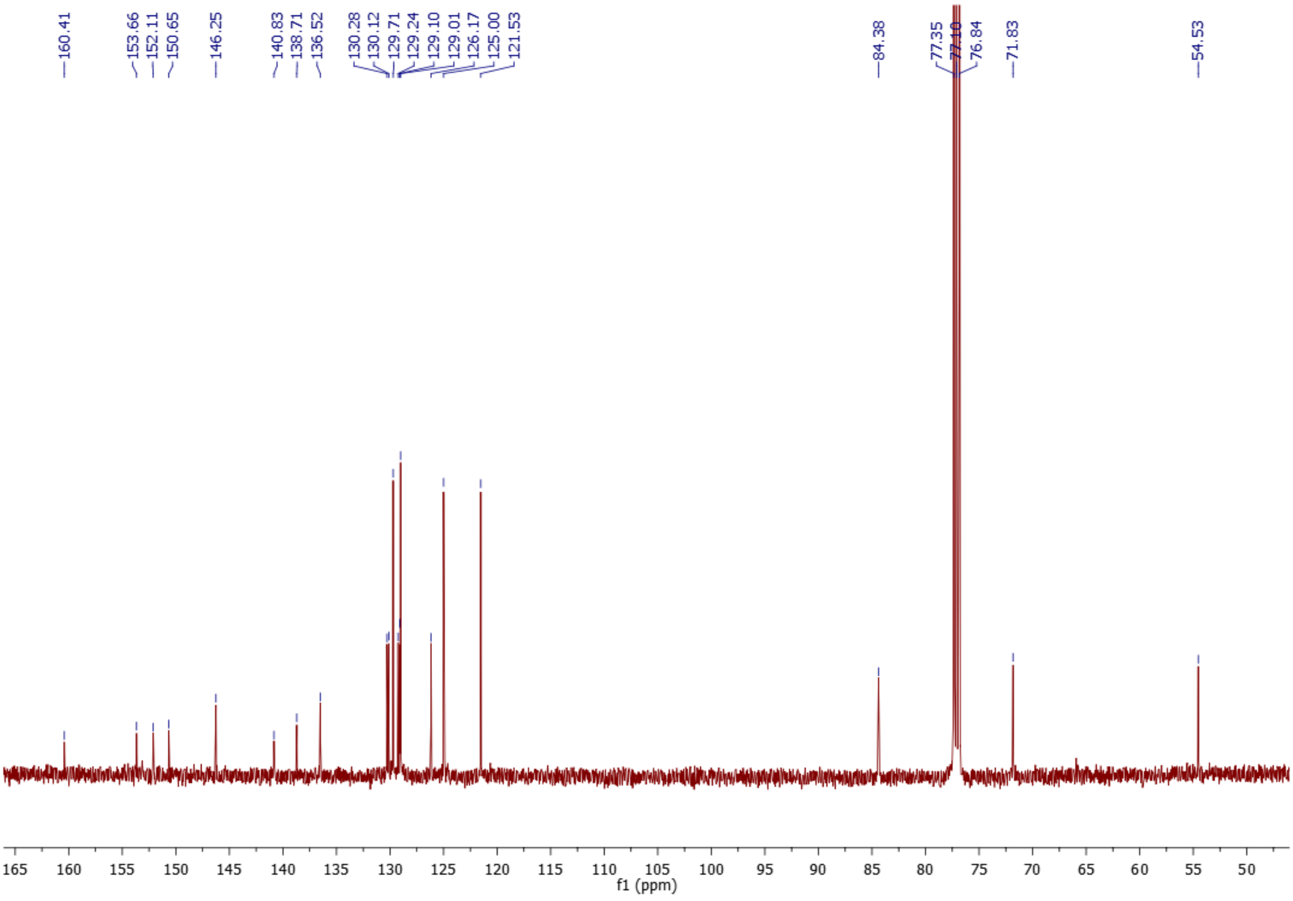
160.41
153.66
152.11
150.65
146.25
140.83
138.71
136.52
130.28
130.12
129.71
129.24
129.10
129.01
126.17
125.00
121.53
84.38
77.35
77.10
76.84
71.83
54.53
165 160 155 150 145 140 135 130 125 120 115 110 105 100 95 90 85 80 75 70 65 60 55 50
f1 (ppm)

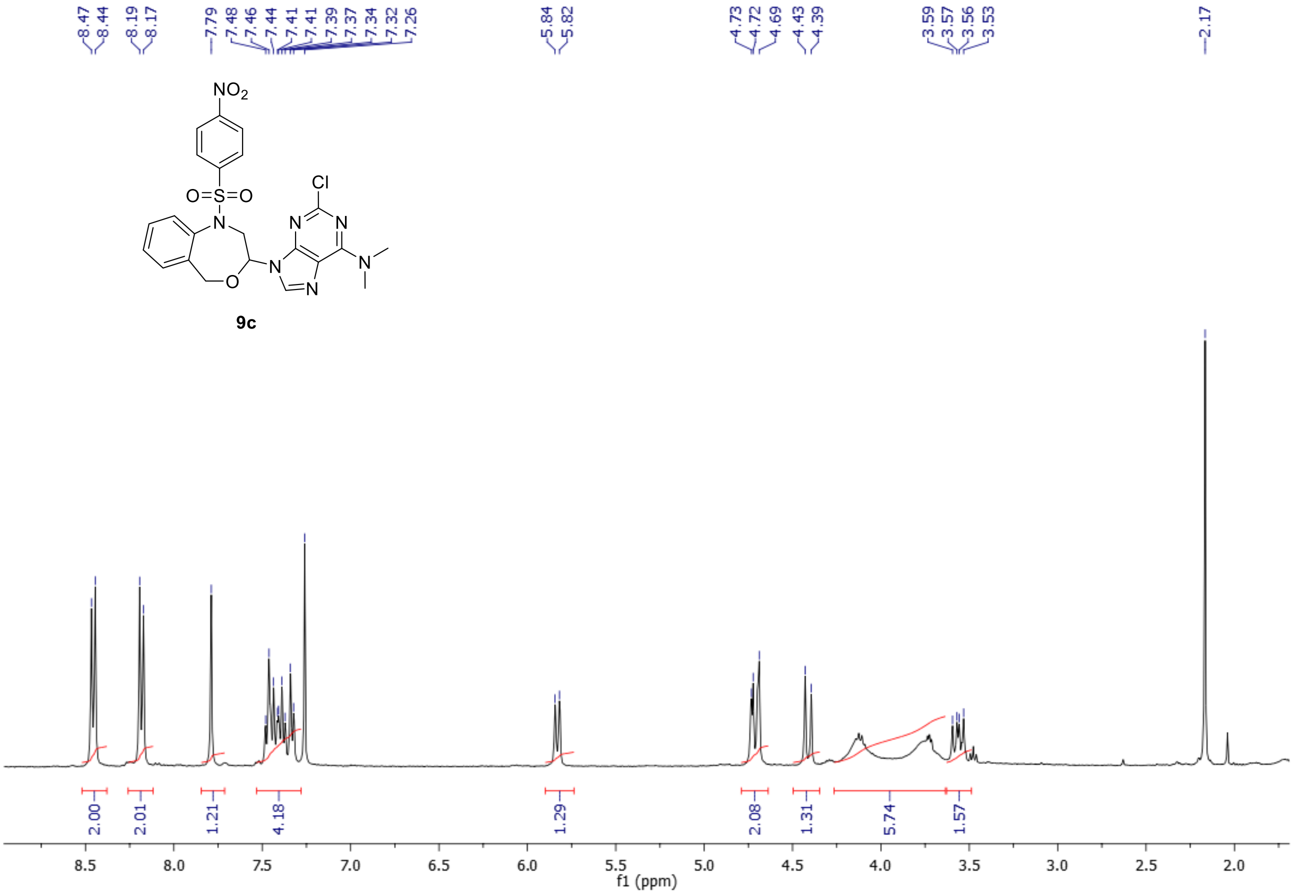
8.47 8.44 8.19 8.17 7.79 7.48 7.46 7.44 7.41 7.41 7.39 7.37 7.34 7.32 7.26 5.84 5.82 4.73 4.72 4.69 4.43 4.39 3.59 3.57 3.56 3.53 2.17
NO_2
O=S=O
N
O
Cl
N
N
N
N
9c
2.00 2.01 1.21 4.18 1.29 2.08 1.31 5.74 1.57
f1 (ppm)

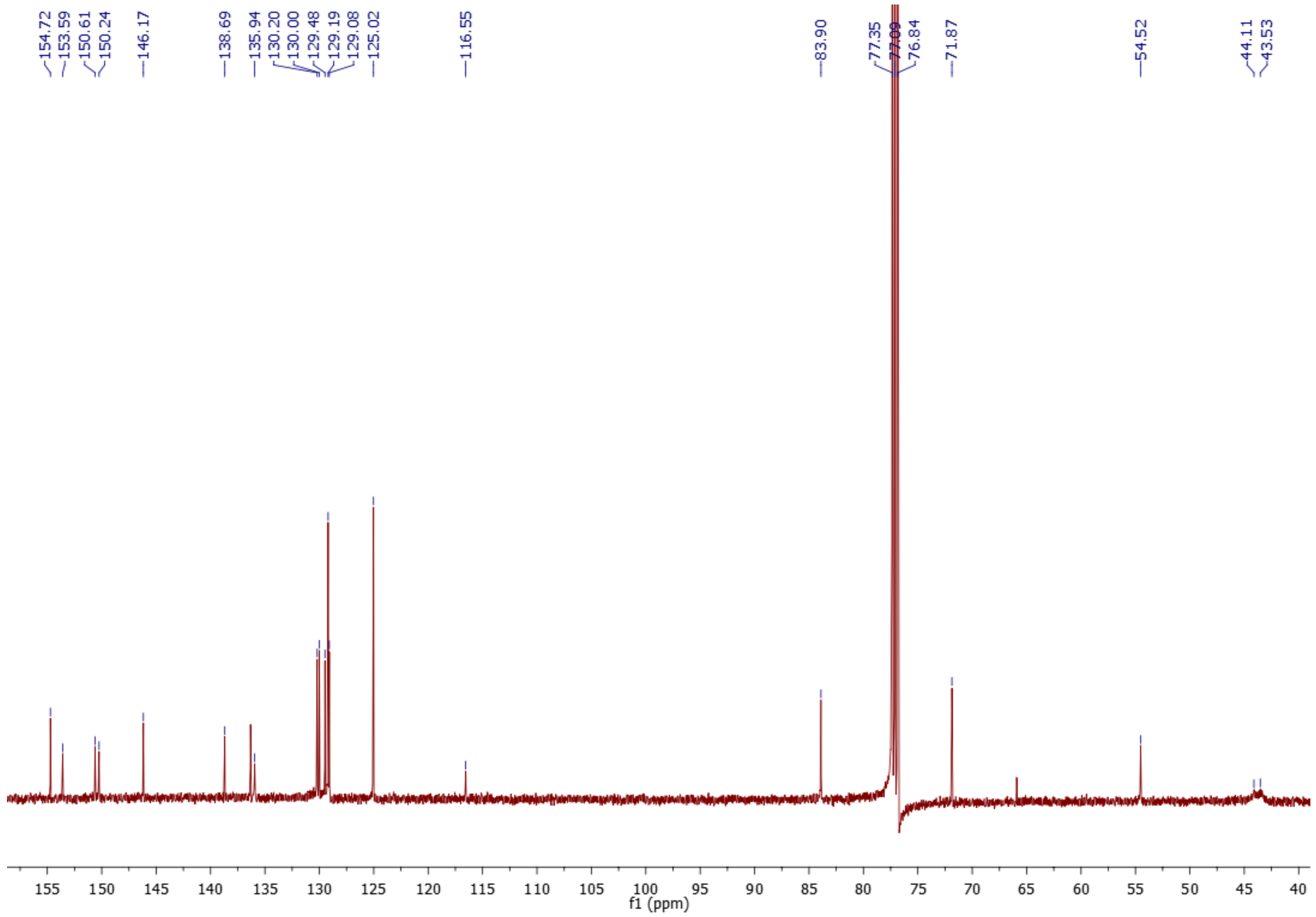
154.72 153.59 150.61 150.24 146.17 138.69 135.94 130.20 130.00 129.48 129.19 129.08 125.02 116.55 83.90 77.35 77.09 76.84 71.87 54.52 44.11 43.53
f1 (ppm)

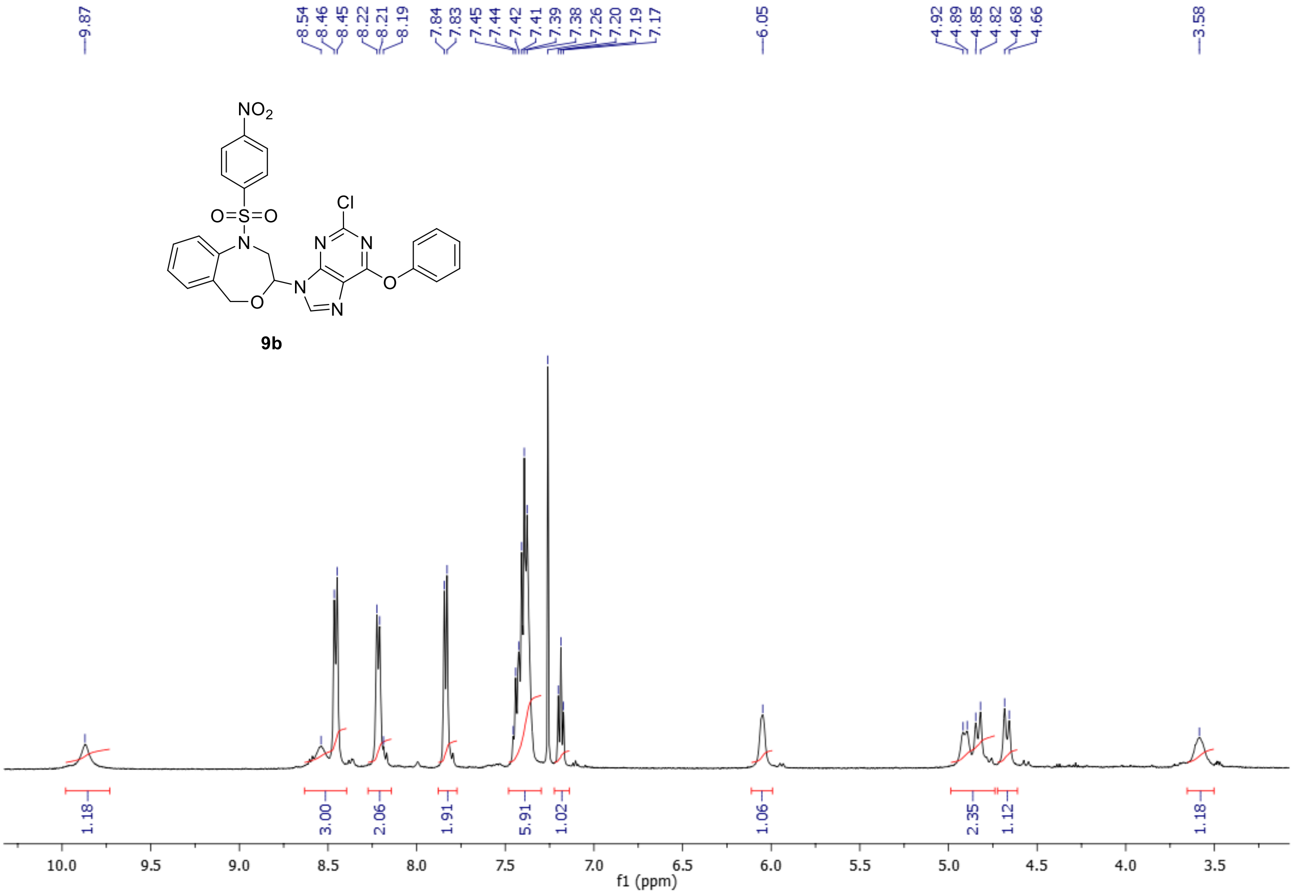
9.87
8.54
8.46
8.45
8.22
8.21
8.19
7.84
7.83
7.45
7.44
7.42
7.41
7.39
7.38
7.26
7.20
7.19
7.17
6.05
4.92
4.89
4.85
4.82
4.68
4.66
3.58
NO2
Cl
N
O=S=O
O
9b
1.18
3.00
2.06
1.91
5.91
1.02
1.06
2.35
1.12
1.18
10.0
9.5
9.0
8.5
8.0
7.5
7.0
6.5
6.0
5.5
5.0
4.5
4.0
3.5
f1 (ppm)

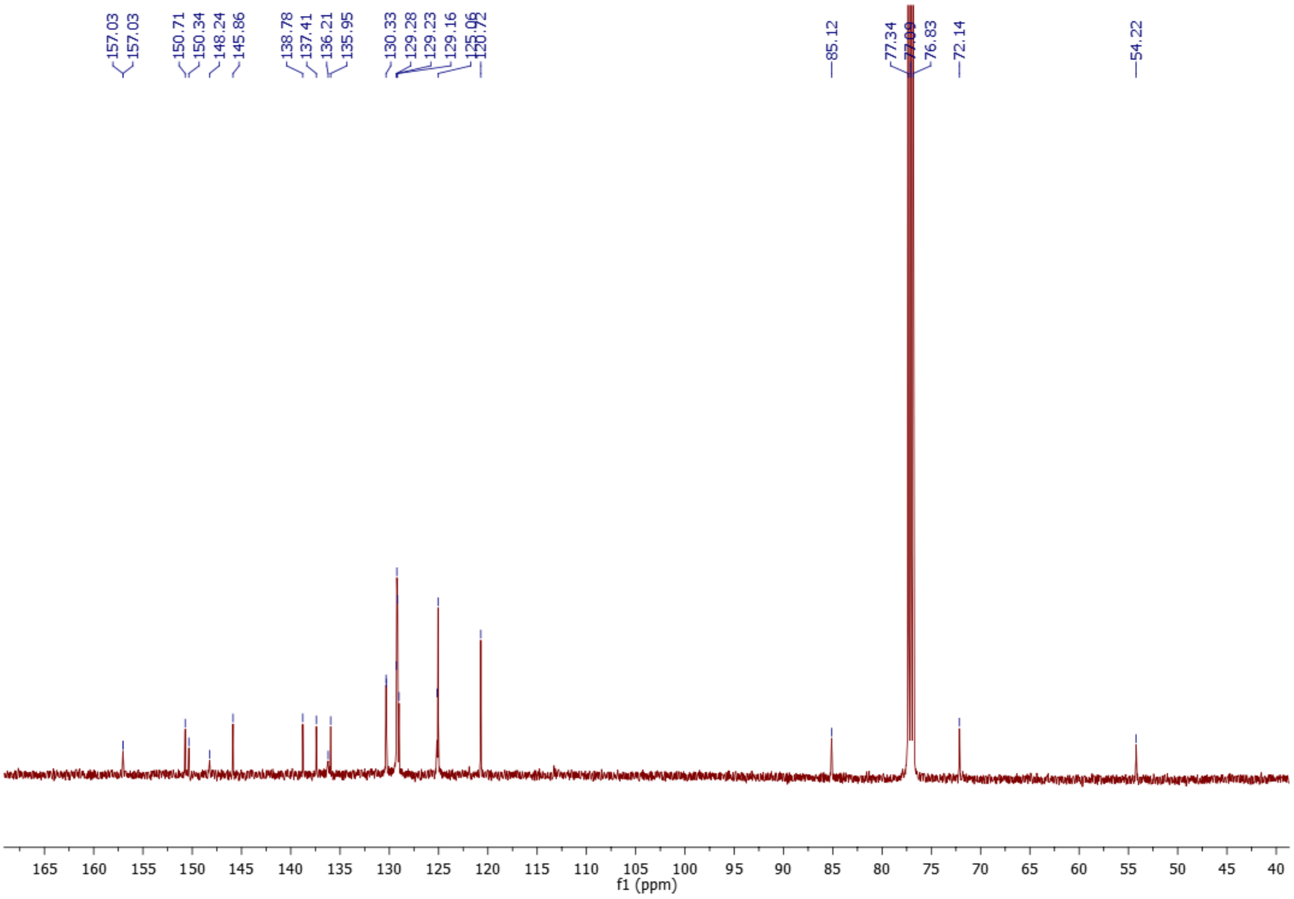
157.03
157.03
150.71
150.34
148.24
145.86
138.78
137.41
136.21
135.95
130.33
129.28
129.23
129.16
125.06
120.72
85.12
77.34
77.09
76.83
72.14
54.22
165
160
155
150
145
140
135
130
125
120
115
110
105
100
95
90
85
80
75
70
65
60
55
50
45
40
f1 (ppm)

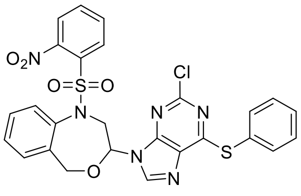
O2N
O=S=O
N
Cl
N
N
S
N
N
O

9e

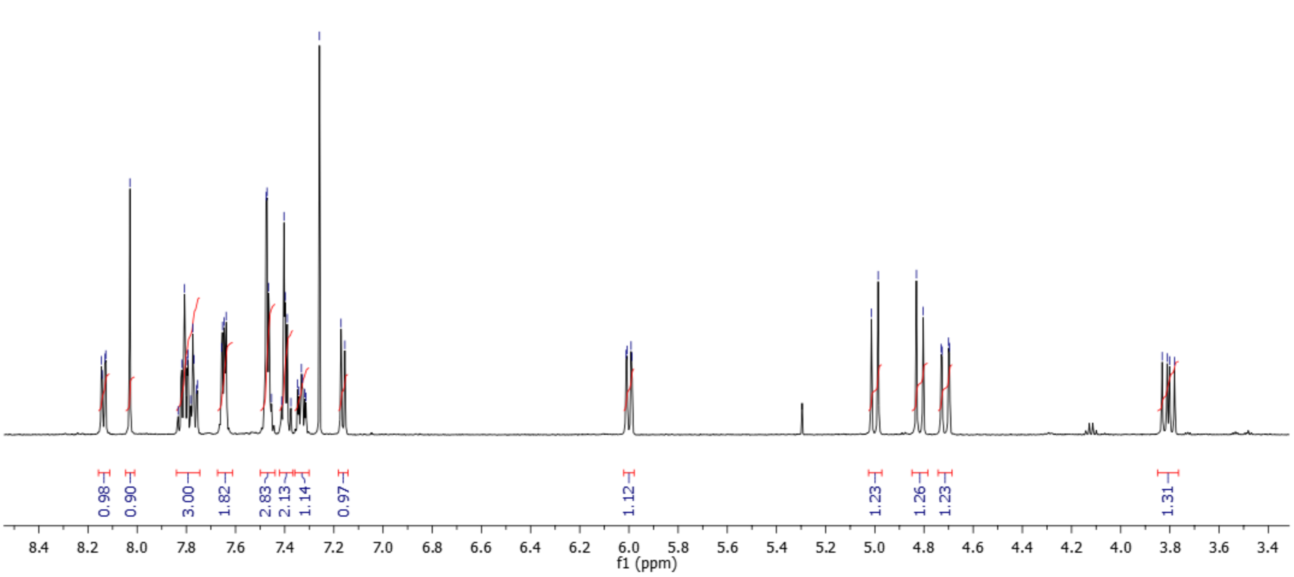
8.15
8.14
8.13
8.13
8.03
7.82
7.81
7.80
7.79
7.77
7.77
7.66
7.65
7.65
7.64
7.48
7.47
7.47
7.40
7.39
7.33
7.33
7.26
7.17
7.16
6.01
6.01
5.99
5.99
5.01
4.99
4.83
4.80
4.73
4.73
4.70
4.70
3.83
3.81
3.80
3.78
0.98
0.90
3.00
1.82
2.83
2.13
1.14
0.97
1.12
1.23
1.26
1.23
1.31
8.4
8.2
8.0
7.8
7.6
7.4
7.2
7.0
6.8
6.6
6.4
6.2
6.0
5.8
5.6
5.4
5.2
5.0
4.8
4.6
4.4
4.2
4.0
3.8
3.6
3.4
f1 (ppm)

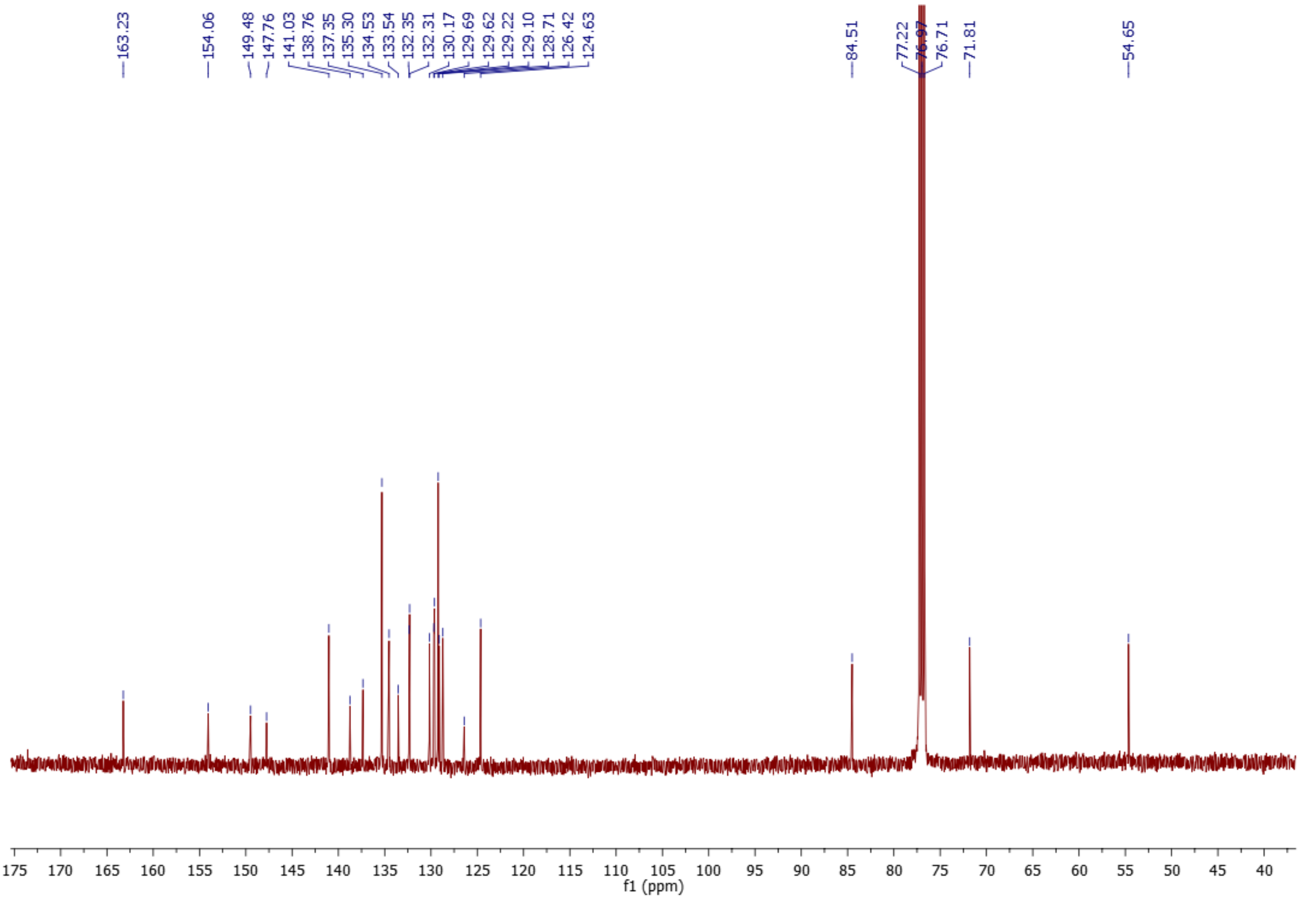
163.23
154.06
149.48
147.76
141.03
138.76
137.35
135.30
134.53
133.54
132.35
132.31
130.17
129.69
129.62
129.22
129.10
128.71
126.42
124.63
84.51
77.22
76.97
76.71
71.81
54.65
175
170
165
160
155
150
145
140
135
130
125
120
115
110
105
100
95
90
85
80
75
70
65
60
55
50
45
40
f1 (ppm)

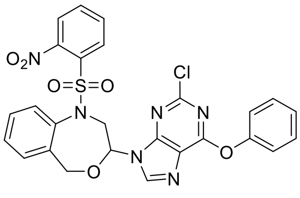

9f

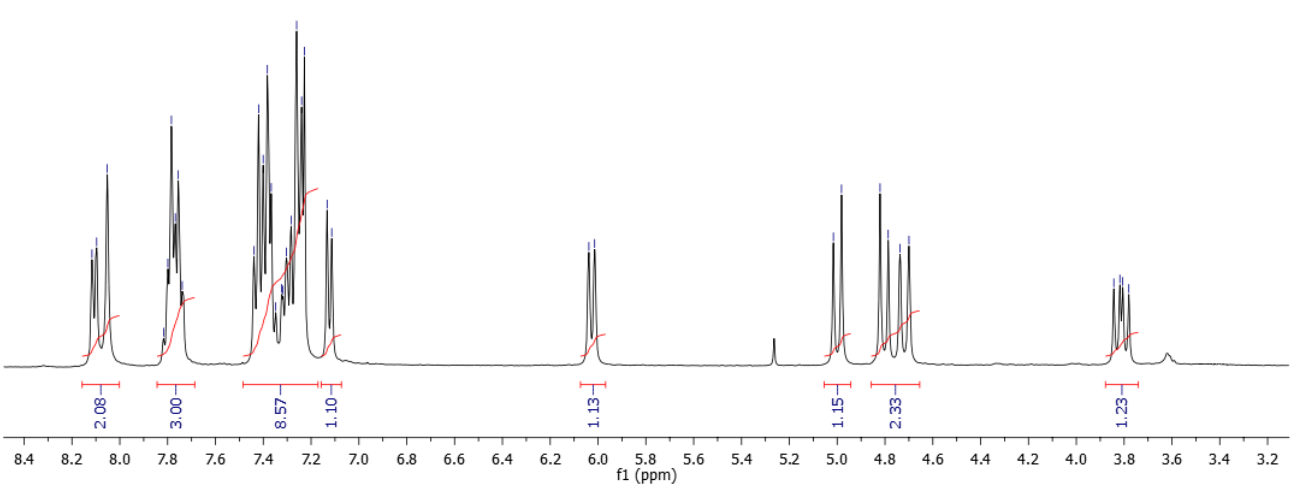

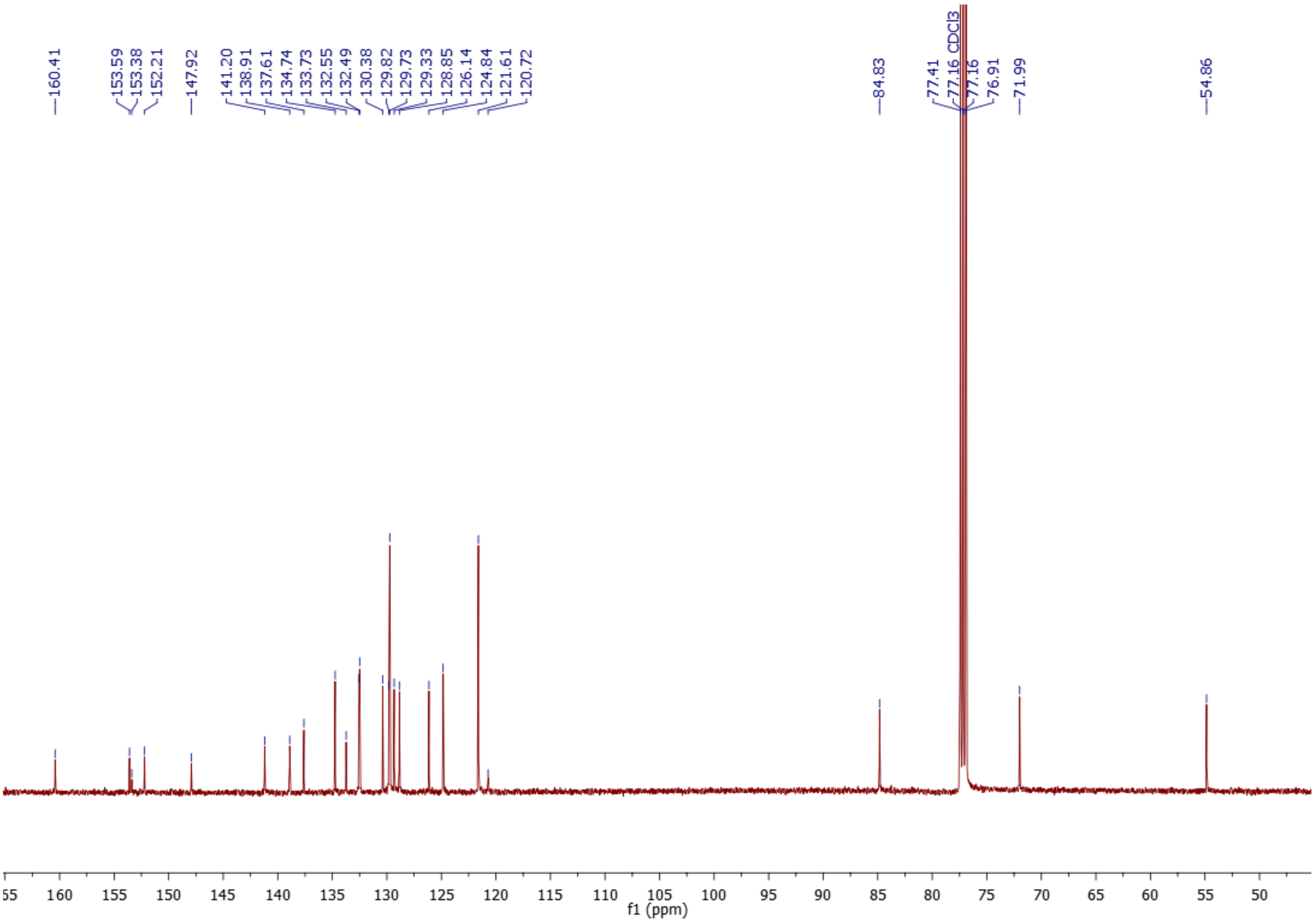

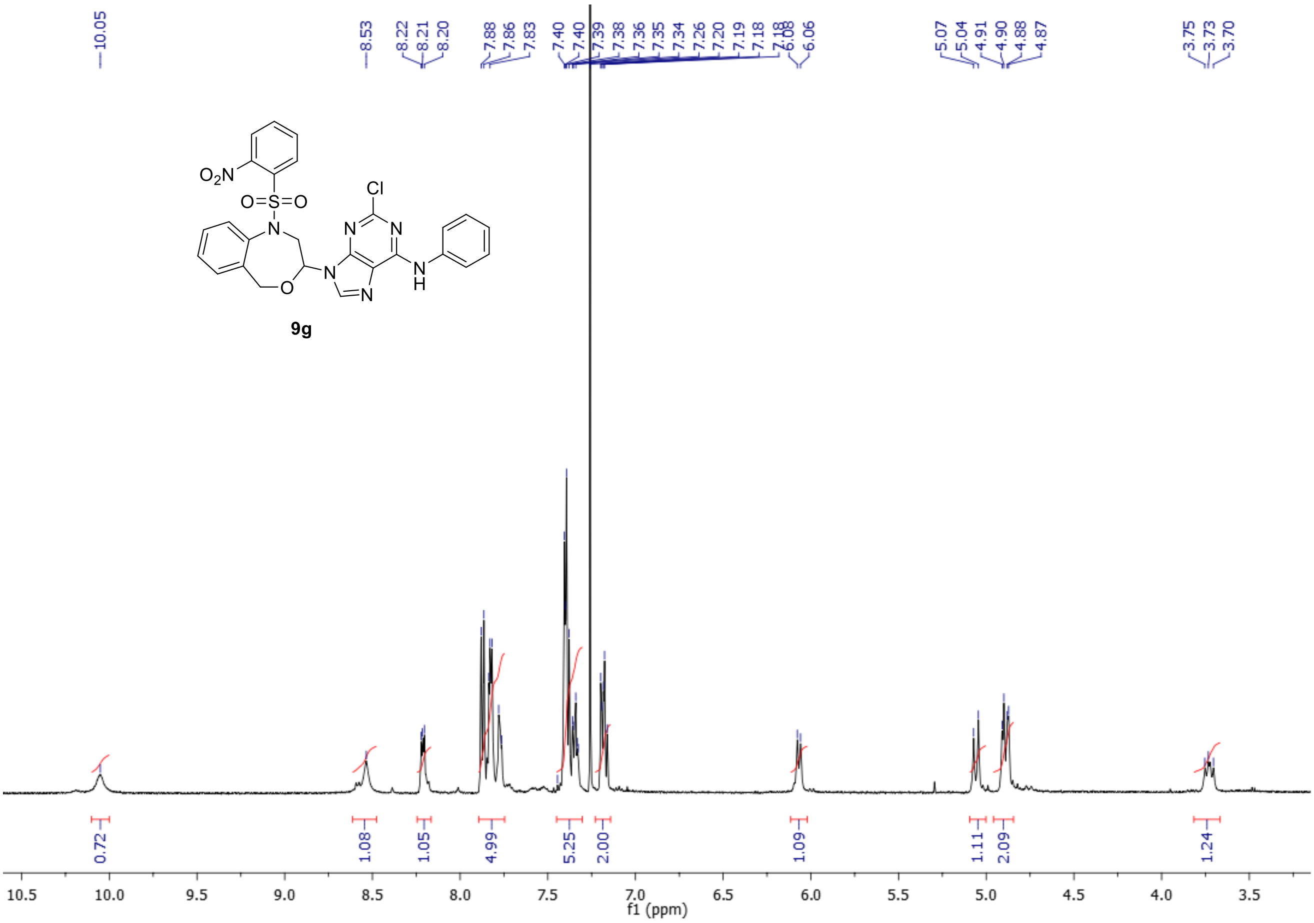
10.05
8.53
8.22
8.21
8.20
7.88
7.86
7.83
7.40
7.40
7.39
7.38
7.36
7.35
7.34
7.26
7.20
7.19
7.18
7.18
6.08
6.06
5.07
5.04
4.91
4.90
4.88
4.87
3.75
3.73
3.70
9g
0.72
1.08
1.05
4.99
5.25
2.00
1.09
1.11
2.09
1.24
f1 (ppm)

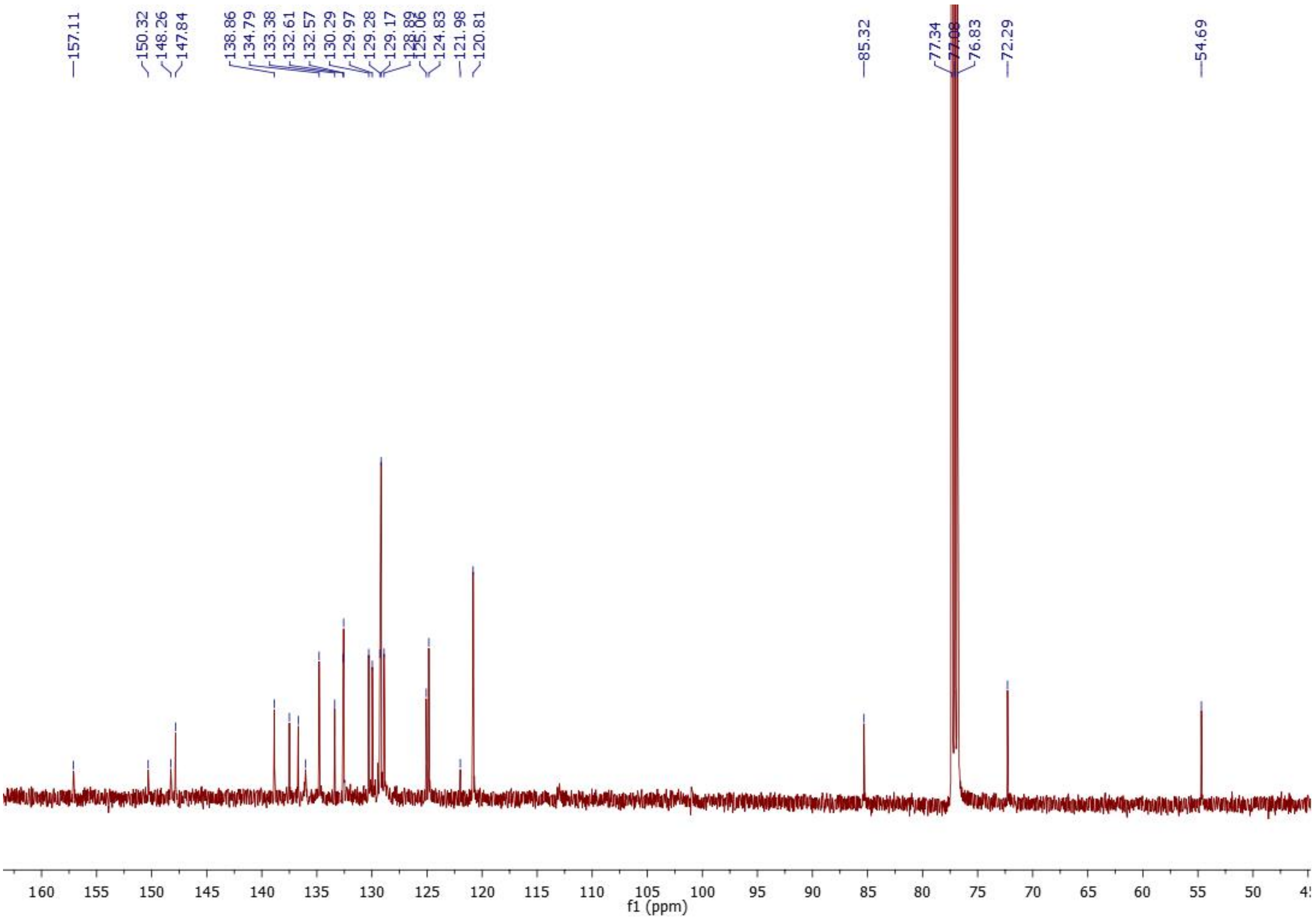
157.11
150.32
148.26
147.84
138.86
134.79
133.38
132.61
132.57
130.29
129.97
129.28
129.17
125.06
124.83
121.98
120.81
85.32
77.34
76.83
72.29
54.69
f1 (ppm)

2. Mass Spectra

Compound 7a

Elemental Composition Report **Page 1**

Single Mass Analysis
Tolerance = 10.0 PPM / DBE: min = -1.5, max = 50.0
Element prediction: Off
Number of isotope peaks used for i-FIT = 3

Monoisotopic Mass, Even Electron Ions
234 formula(e) evaluated with 1 results within limits (up to 50 best isotopic matches for each mass)
Elements Used:
C: 0-23 H: 0-1000 N: 0-5 O: 0-5 S: 0-1 Cl: 0-1
OC148 3 (0.070) AM (Top,1, Ht,5000.0,0.00,1.00)
1: TOF MS ES+

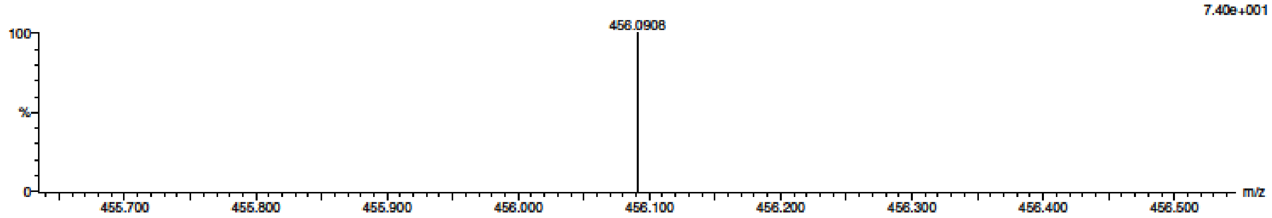

Minimum:				-1.5			
Maximum:		5.0	10.0	50.0			
Mass	Calc. Mass	mDa	PPM	DBE	i-FIT	i-FIT (Norm)	Formula
456.0908	456.0897	1.1	2.4	14.5	18.8	0.0	C21 H19 N5 O3 S Cl

Compound 7b

Elemental Composition Report **Page 1**

Single Mass Analysis
Tolerance = 10.0 PPM / DBE: min = -1.5, max = 50.0
Element prediction: Off
Number of isotope peaks used for i-FIT = 3

Monoisotopic Mass, Even Electron Ions
498 formula(e) evaluated with 2 results within limits (up to 50 best isotopic matches for each mass)
Elements Used:
C: 0-21 H: 0-1000 N: 0-9 O: 0-3 Na: 0-1 S: 0-1 Br: 0-1

OC-317 3 (0.036) AM (Cen,6, 100.00, Ht,5000.0,0.00,1.00); Cm (2:5)
1: TOF MS ES+

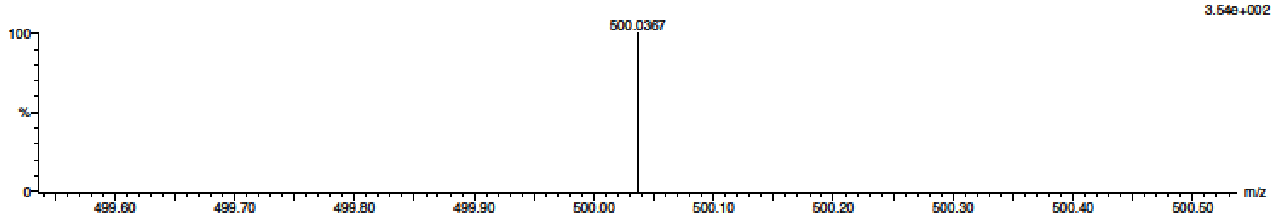

Minimum:				-1.5			
Maximum:		5.0	10.0	50.0			
Mass	Calc. Mass	mDa	PPM	DBE	i-FIT	i-FIT (Norm)	Formula
500.0367	500.0392	-2.5	-5.0	14.5	23.3	0.8	C21 H19 N5 O3 S Br
	500.0368	-0.1	-0.2	11.5	23.1	0.6	C19 H20 N5 O3 Na S Br

Compound 7c

Elemental Composition Report **Page 1**

Single Mass Analysis
Tolerance = 10.0 PPM / DBE: min = -1.5, max = 50.0
Element prediction: Off
Number of isotope peaks used for i-FIT = 3

Monoisotopic Mass, Even Electron Ions
309 formula(e) evaluated with 1 results within limits (up to 50 best isotopic matches for each mass)
Elements Used:
C: 0-23 H: 0-1000 N: 0-5 O: 0-5 S: 0-1 Cl: 0-2
MN128 6 (0.107) AM (Top,6, Ht,5000.0,0.00,1.00); Cm (5:9)
1: TOF MS ES+

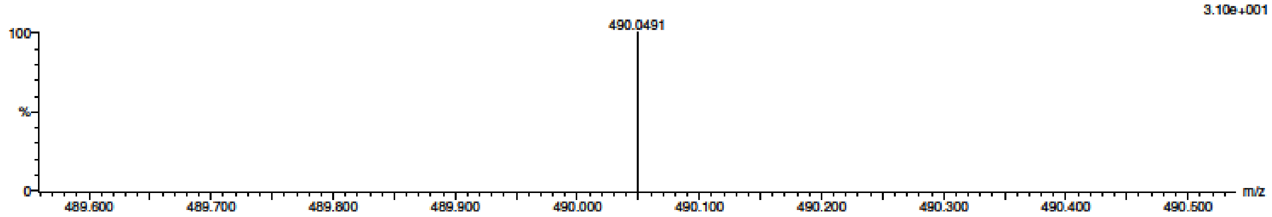

Minimum:				-1.5			
Maximum:		5.0	10.0	50.0			
Mass	Calc. Mass	mDa	PPM	DBE	i-FIT	i-FIT (Norm)	Formula
490.0491	490.0507	-1.6	-3.3	14.5	17.4	0.0	C21 H18 N5 O3 S Cl2

Compound 8

Elemental Composition Report **Page 1**

Single Mass Analysis
Tolerance = 10.0 PPM / DBE: min = -1.5, max = 50.0
Element prediction: Off
Number of isotope peaks used for i-FIT = 3

Monoisotopic Mass, Even Electron Ions
1457 formula(e) evaluated with 8 results within limits (up to 50 best isotopic matches for each mass)
Elements Used:
C: 0-21 H: 0-200 N: 0-5 O: 0-5 Na: 0-1 S: 0-2 I: 0-3

OC-28 113 (0.377) TOF MS ES+
6.50e+001

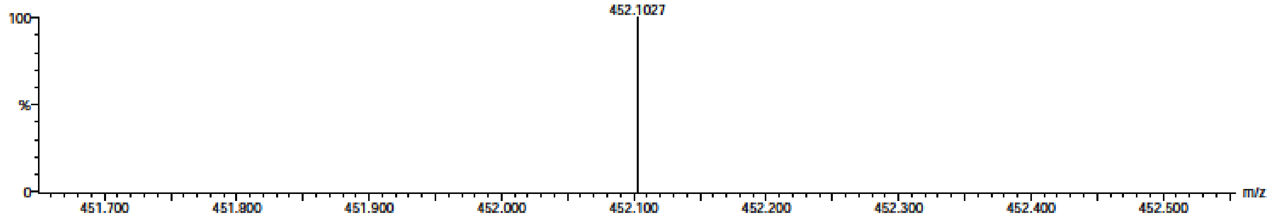

Minimum:				-1.5			
Maximum:		5.0	10.0	50.0			
Mass	Calc. Mass	mDa	PPM	DBE	i-FIT	i-FIT (Norm)	Formula
452.1027	452.1029	-0.2	-0.4	15.5	17.6	2.3	C21 H18 N5 O5 S
	452.1063	-3.6	-8.0	5.5	17.7	2.5	C19 H28 N O2 Na I
	452.1005	2.2	4.9	12.5	17.6	2.4	C19 H19 N5 O5 Na S
	452.1062	-3.5	-7.7	10.5	17.9	2.7	C18 H22 N5 O5 S2
	452.1046	-1.9	-4.2	4.5	17.2	1.9	C16 H27 N3 O4 I
	452.1038	-1.1	-2.4	7.5	17.4	2.1	C16 H23 N5 O5 Na S2
	452.1022	0.5	1.1	1.5	16.8	1.5	C14 H28 N3 O4 Na I
	452.1015	1.2	2.7	-0.5	17.0	1.7	C12 H31 N5 O S2 I

Compound 9a

Elemental Composition Report **Page 1**

Single Mass Analysis
Tolerance = 20.0 PPM / DBE: min = -1.5, max = 50.0
Element prediction: Off
Number of isotope peaks used for i-FIT = 3

Monoisotopic Mass, Even Electron Ions
264 formula(e) evaluated with 1 results within limits (up to 50 best isotopic matches for each mass)
Elements Used:
C: 0-26 H: 0-1000 N: 0-6 O: 0-5 S: 0-2 Cl: 0-1
MN39F1 6 (0.107) AM (Top,1, Ht,5000.0,0.00,1.00)
1: TOF MS ES+

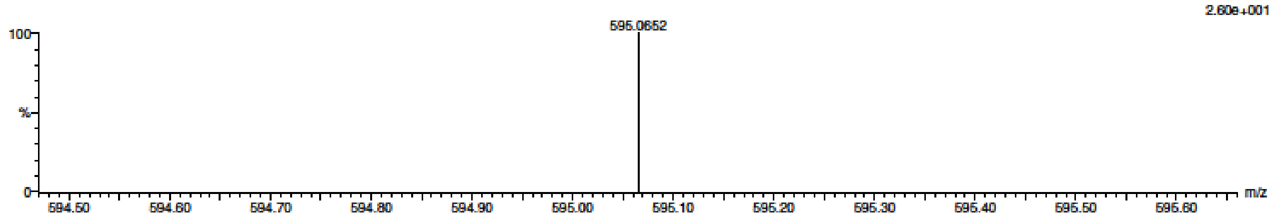

	Mass	Calc. Mass	mDa	PPM	DBE	i-FIT	i-FIT (Norm)	Formula
Minimum:					-1.5			
Maximum:			5.0	20.0	50.0			
	595.0652	595.0625	2.7	4.5	19.5	15.9	0.0	C26 H20 N6 O5 S2 Cl

Compound 9b

Elemental Composition Report **Page 1**

Single Mass Analysis
Tolerance = 20.0 PPM / DBE: min = -1.5, max = 50.0
Element prediction: Off
Number of isotope peaks used for i-FIT = 3

Monoisotopic Mass, Even Electron Ions
350 formula(e) evaluated with 2 results within limits (up to 50 best isotopic matches for each mass)
Elements Used:
C: 0-26 H: 0-1000 N: 0-6 O: 0-6 S: 0-2 Cl: 0-1
MN37F3bF1 7 (0.124) AM (Top,1, Ht,5000.0,0.00,1.00); Cm (7)
1: TOF MS ES+

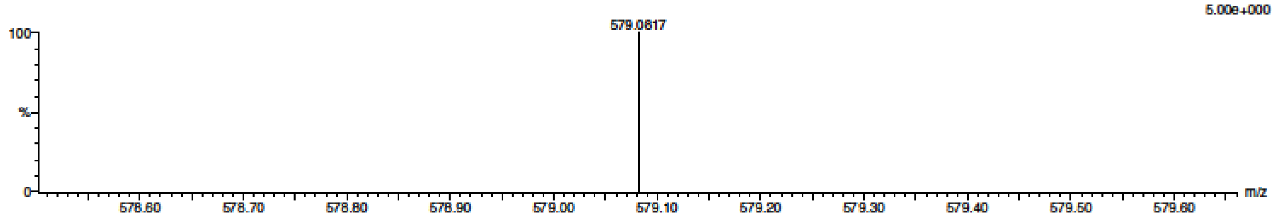

	Mass	Calc. Mass	mDa	PPM	DBE	i-FIT	i-FIT (Norm)	Formula
Minimum:					-1.5			
Maximum:			5.0	20.0	50.0			
	579.0817	579.0854	-3.7	-6.4	19.5	12.2	0.6	C26 H20 N6 O6 S Cl
		579.0887	-7.0	-12.1	14.5	12.4	0.8	C23 H24 N6 O6 S2 Cl

Compound 9c

Elemental Composition Report | **Page 1**

Single Mass Analysis
Tolerance = 20.0 PPM / DBE: min = -1.5, max = 50.0
Element prediction: Off
Number of isotope peaks used for i-FIT = 3

Monoisotopic Mass, Even Electron Ions
402 formula(e) evaluated with 4 results within limits (up to 50 best isotopic matches for each mass)
Elements Used:
C: 0-22 H: 0-1000 N: 0-7 O: 0-6 S: 0-2 Cl: 0-1
MN37F2b 4 (0.053) AM (Top,1, Ht,5000.0,0.00,1.00)
1: TOF MS ES+

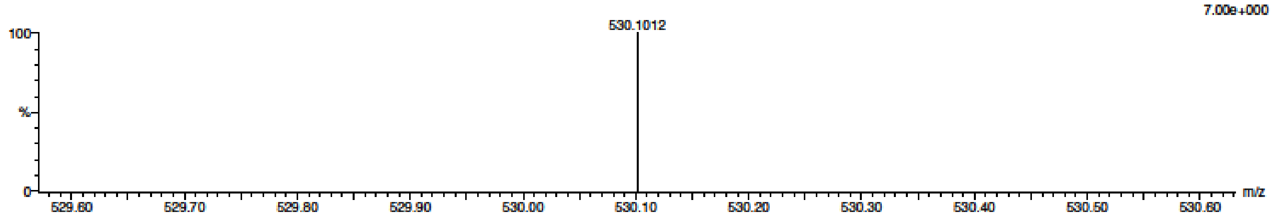

Minimum:				-1.5			
Maximum:		5.0	20.0	50.0			
Mass	Calc. Mass	mDa	PPM	DBE	i-FIT	i-FIT (Norm)	Formula
530.1012	530.1013	-0.1	-0.2	15.5	12.4	1.3	C22 H21 N7 O5 S Cl
	530.0917	9.5	17.9	15.5	12.2	1.2	C21 H20 N7 O6 S2
	530.0935	7.7	14.5	10.5	12.7	1.6	C20 H25 N5 O6 S2 Cl
	530.1047	-3.5	-6.6	10.5	12.5	1.5	C19 H25 N7 O5 S2 Cl

Compound 9d

Elemental Composition Report | **Page 1**

Single Mass Analysis
Tolerance = 20.0 PPM / DBE: min = -1.5, max = 50.0
Element prediction: Off
Number of isotope peaks used for i-FIT = 3

Monoisotopic Mass, Even Electron Ions
435 formula(e) evaluated with 4 results within limits (up to 50 best isotopic matches for each mass)
Elements Used:
C: 0-26 H: 0-1000 N: 0-7 O: 0-6 S: 0-2 Cl: 0-1
OC64 5 (0.070) AM (Top,1, Ht,5000.0,0.00,1.00); Cm (5)
1: TOF MS ES+

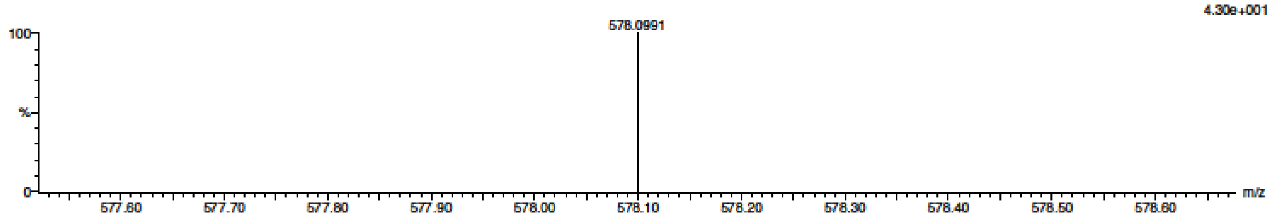

Minimum:				-1.5			
Maximum:		5.0	20.0	50.0			
Mass	Calc. Mass	mDa	PPM	DBE	i-FIT	i-FIT (Norm)	Formula
578.0991	578.1013	-2.2	-3.8	19.5	17.1	1.2	C26 H21 N7 O5 S Cl
	578.0917	7.4	12.8	19.5	17.1	1.3	C25 H20 N7 O6 S2
	578.1047	-5.6	-9.7	14.5	17.4	1.5	C23 H25 N7 O5 S2 Cl
	578.0935	5.6	9.7	14.5	17.4	1.6	C24 H25 N5 O6 S2 Cl

Compound 9e

Elemental Composition Report **Page 1**

Single Mass Analysis
Tolerance = 20.0 PPM / DBE: min = -1.5, max = 50.0
Element prediction: Off
Number of isotope peaks used for i-FIT = 3

Monoisotopic Mass, Even Electron Ions
392 formula(e) evaluated with 1 results within limits (up to 50 best isotopic matches for each mass)
Elements Used:
C: 0-26 H: 0-1000 N: 0-7 O: 0-6 S: 0-2 Cl: 0-1
MN27 5 (0.070) AM (Top,1, Ht,5000.0,0.00,1.00)
1: TOF MS ES+

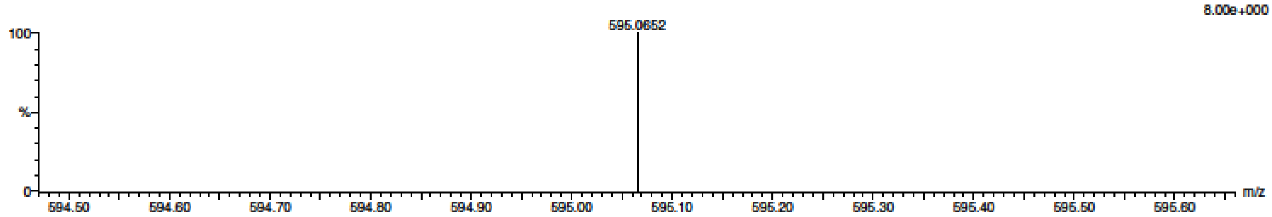

Minimum:				-1.5			
Maximum:		5.0	20.0	50.0			
Mass	Calc. Mass	mDa	PPM	DBE	i-FIT	i-FIT (Norm)	Formula
595.0652	595.0625	2.7	4.5	19.5	12.9	0.0	C26 H20 N6 O5 S2 Cl

Compound 9f

Elemental Composition Report **Page 1**

Single Mass Analysis
Tolerance = 20.0 PPM / DBE: min = -1.5, max = 50.0
Element prediction: Off
Number of isotope peaks used for i-FIT = 3

Monoisotopic Mass, Even Electron Ions
435 formula(e) evaluated with 2 results within limits (up to 50 best isotopic matches for each mass)
Elements Used:
C: 0-26 H: 0-1000 N: 0-7 O: 0-6 S: 0-2 Cl: 0-1
MN35bF1 5 (0.070) AM (Top,1, Ht,5000.0,0.00,1.00); Cm (5)
1: TOF MS ES+

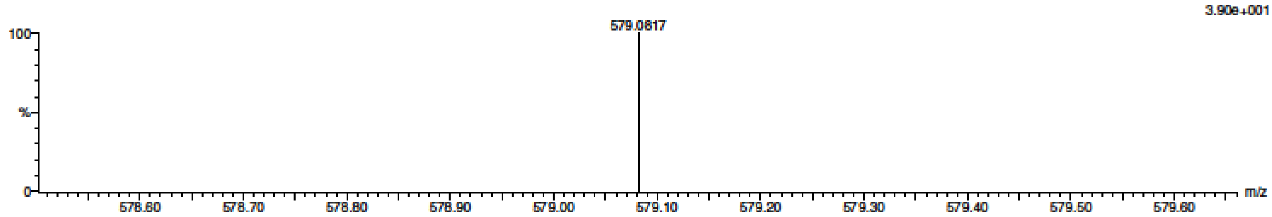

Minimum:				-1.5			
Maximum:		5.0	20.0	50.0			
Mass	Calc. Mass	mDa	PPM	DBE	i-FIT	i-FIT (Norm)	Formula
579.0817	579.0854	-3.7	-6.4	19.5	16.9	0.5	C26 H20 N6 O6 S Cl
	579.0887	-7.0	-12.1	14.5	17.3	0.9	C23 H24 N6 O6 S2 Cl

Compound 9g

Elemental Composition Report **Page 1**

Single Mass Analysis
Tolerance = 20.0 PPM / DBE: min = -1.5, max = 50.0
Element prediction: Off
Number of isotope peaks used for i-FIT = 3

Monoisotopic Mass, Even Electron Ions
435 formula(e) evaluated with 4 results within limits (up to 50 best isotopic matches for each mass)
Elements Used:
C: 0-26 H: 0-1000 N: 0-7 O: 0-6 S: 0-2 Cl: 0-1
MN34F3 69 (1.479) AM (Top,1, Ht,5000.0,0.00,1.00)
1: TOF MS ES+

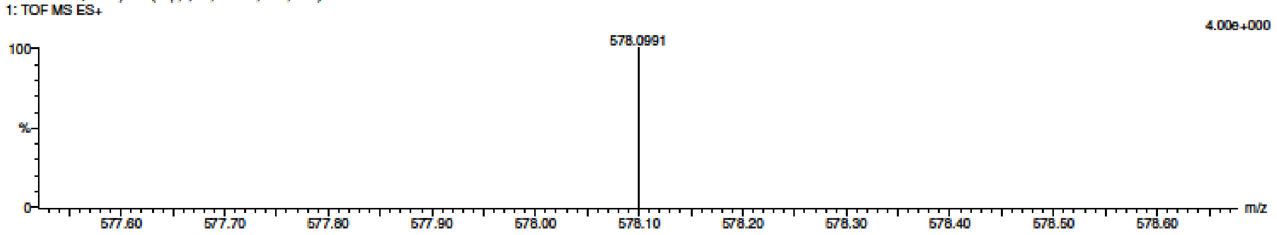

Minimum:				-1.5			
Maximum:		5.0	20.0	50.0			

Mass	Calc. Mass	mDa	PPM	DBE	i-FIT	i-FIT (Norm)	Formula
578.0991	578.1013	-2.2	-3.8	19.5	12.1	1.3	C26 H21 N7 O5 S Cl
	578.0917	7.4	12.8	19.5	12.2	1.4	C25 H20 N7 O6 S2
	578.1047	-5.6	-9.7	14.5	12.2	1.4	C23 H25 N7 O5 S2 Cl
	578.0935	5.6	9.7	14.5	12.2	1.4	C24 H25 N5 O6 S2 Cl
